# Dynamic human liver proteome atlas reveals functional insights into disease pathways

**DOI:** 10.15252/msb.202210947

**Published:** 2022-05-17

**Authors:** Lili Niu, Philipp E Geyer, Rajat Gupta, Alberto Santos, Florian Meier, Sophia Doll, Nicolai J Wewer Albrechtsen, Sabine Klein, Cristina Ortiz, Frank E Uschner, Robert Schierwagen, Jonel Trebicka, Matthias Mann

**Affiliations:** ^1^ Novo Nordisk Foundation Center for Protein Research Faculty of Health and Medical Sciences University of Copenhagen Copenhagen Denmark; ^2^ Department of Proteomics and Signal Transduction Max Planck Institute of Biochemistry Martinsried Germany; ^3^ Center for Health Data Science Faculty of Health Sciences University of Copenhagen Copenhagen Denmark; ^4^ Big Data Institute Nuffield Department of Medicine University of Oxford Oxford UK; ^5^ Department of Clinical Biochemistry Rigshospitalet University of Copenhagen Copenhagen Denmark; ^6^ Department of Internal Medicine I Goethe University Clinic Frankfurt Frankfurt Germany; ^7^ Department of Internal Medicine B WW University Münster Münster Germany; ^8^ European Foundation for the Study of Chronic Failure, EFCLIF Barcelona Spain; ^9^ Present address: OmicEra Diagnostics GmbH Planegg Germany; ^10^ Present address: Pfizer Worldwide Research and Development San Diego CA USA; ^11^ Present address: Functional Proteomics Jena University Hospital Jena Germany

**Keywords:** clinical proteomics, liver disease, liver fibrosis, MS‐based proteomics, tissue proteome atlas, Methods & Resources, Molecular Biology of Disease, Proteomics

## Abstract

Deeper understanding of liver pathophysiology would benefit from a comprehensive quantitative proteome resource at cell type resolution to predict outcome and design therapy. Here, we quantify more than 150,000 sequence‐unique peptides aggregated into 10,000 proteins across total liver, the major liver cell types, time course of primary cell cultures, and liver disease states. Bioinformatic analysis reveals that half of hepatocyte protein mass is comprised of enzymes and 23% of mitochondrial proteins, twice the proportion of other liver cell types. Using primary cell cultures, we capture dynamic proteome remodeling from tissue states to cell line states, providing useful information for biological or pharmaceutical research. Our extensive data serve as spectral library to characterize a human cohort of non‐alcoholic steatohepatitis and cirrhosis. Dramatic proteome changes in liver tissue include signatures of hepatic stellate cell activation resembling liver cirrhosis and providing functional insights. We built a web‐based dashboard application for the interactive exploration of our resource (www.liverproteome.org).

## Graphical abstract

In‐depth acquisition and analysis of quantitative human liver proteome reveals proteome differences between liver cell types, proteome dynamics during primary cell culture and proteome changes in liver cirrhosis and NASH. This resource can be explored at www.liverproteome.org.

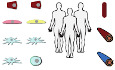

## Introduction

The liver is essential for the human body’s homeostasis and maintains a well‐orchestrated network of parenchymal and non‐parenchymal cell types, interconnecting the vascular and biliary system. While hepatocytes perform key metabolic functions, detoxification, and protein synthesis, the non‐parenchymal cells provide a microenvironment for substance exchange and promote inflammatory and immunological responses (Kmiec, 2001; Shetty *et al*, 2018). The liver is constantly exposed to gut‐derived dietary antigens, microbial products and toxic substances such as alcohol, drugs, and excess lipids, all of which can induce liver damage. Chronic liver injury results in persistent hepatic inflammation, which can further progress to fibrosis and eventually cirrhosis—the common end‐stage of chronic liver disease (CLD). CLD—including alcohol‐related and non‐alcoholic fatty liver disease (ALD and NAFLD)—is a major global health problem affecting approximately 1.5 billion people and causing more than two million deaths annually due to complications of cirrhosis and hepatocellular carcinoma (Loomba & Sanyal, 2013; Asrani *et al*, 2019; Moon *et al*, 2020). Liver disease is also important to study as a comorbidity, which limits or precludes effective treatment of extrahepatic diseases such as malignancy (Mokdad *et al*, 2014; Gu *et al*, 2022). Combined with its typically silent progression, there is an urgent need to implement screening programs in at‐risk populations for early diagnosis (Ginès *et al*, 2016; Marcellin & Kutala, 2018; Collaborators, 2020). Existing tests have limited performance, especially at detecting early disease stages that are still reversible. Thus, biomarker discovery is an active research area and is ideally complemented by underlying biological mechanisms by which markers indicate disease states. This requires knowledge of the tissue and cellular origin of the detected abnormal protein levels and the biological pathways through which the protein plays a role in disease. In this regard, we recently investigated paired plasma and liver biopsies in a large ALD cohort, connecting circulating markers to tissue proteome changes, providing additional validity (preprint: Niu *et al*, 2020). However, we still lack information at the cell type level which would greatly help interpret results from plasma or bulk liver tissue proteomics analysis.

As the main parenchymal cells, hepatocytes, despite their great regenerative capacity, may undergo fatty degeneration and release damage‐associated molecular patterns (DAMPs) to promote chronic inflammation and even transform to malignant cells during CLD. The resident hepatic macrophages (Kupffer cells) are crucial in the pathogenesis of chronic and acute liver diseases, orchestrating both the resolution and progression of inflammation and tissue repair (Krenkel & Tacke, 2017; Lefere & Tacke, 2019; Wen *et al*, 2021). Hepatic stellate cells (HSCs) play a key role in the subsequent development of fibrosis (Mederacke *et al*, 2013). Upon liver injury, HSCs transdifferentiate from vitamin A‐storing and quiescent HSCs to proliferative, fibrogenic, myofibroblast‐like cells—a process termed “HSC activation”. While it is often difficult to isolate activated HSCs in patients, activation can also be induced in primary cell culture. Characterizing the proteome dynamics of this process may lead to new antifibrotic therapeutic options as most drug targets are proteins. Furthermore, such proteome shift upon primary cell culture reports the extent to which it reflects *in vivo* conditions (Pan *et al*, 2009; Azimifar *et al*, 2014; Heslop *et al*, 2017).

Mass spectrometry (MS)‐based proteomics enables global and targeted analysis of proteins in a systematic, systems‐wide and quantitative fashion (Aebersold & Mann, 2016). Although important insights into human liver proteomes have been generated by international efforts (Sun *et al*, 2010; Kampf *et al*, 2014), due to the nature of the technologies used, quantitative readout at the protein level was not sufficient to capture biological or pathological proteome dynamics.

Given the importance of liver pathophysiology, we reasoned that dramatic advances in MS‐based proteomics technologies could provide much more detailed insights into the quantitative human liver proteome. In particular, we wished to use the “proteomic ruler” approach to draw quantitative cellular proteome maps by estimating copy numbers of individual proteins per cell, organelle or pathway given the fixed relation of core histones to DNA in a cell (Wisniewski *et al*, 2014). Here, we performed MS‐based proteomics with fractionation and label‐free quantification on four major primary liver cell types derived from three individuals with normal liver histology, as well as liver biopsy and paired extrahepatic vessels (hepatic artery and portal vein) derived from six individuals undergoing liver transplantation. In‐depth proteomic profiling and analysis revealed “cell‐type specific” expression patterns as well as fundamental differences in cellular proteomes which we interpret in light of anticipated functional specialization. With our extensive dataset, we built a spectral library for single‐shot liver tissue proteomics analysis. Taking advantage of the phased spectrum deconvolution method (ΦSDM; Grinfeld *et al*, 2017) and employing our MS‐interfacing software called MaxQuant.Live (Wichmann *et al*, 2019), we developed an acquisition method which enabled us to achieve deep proteome coverage in only 100 min measurement time. Applying this workflow to a human cohort of non‐alcoholic steatohepatitis (NASH) and liver cirrhosis revealed dramatic proteome changes involving extracellular matrix remodeling, signaling, and metabolic pathways in liver cirrhosis.

We also aimed to capture proteome dynamics during primary cell culture. Time course measurements revealed proteome shifts over time. When integrating results with the human study, we observed that the proteomic signatures of HSC activation largely overlapped with that of liver cirrhosis, providing insights into the cellular origin of the observed proteome changes in bulk cirrhotic liver. We have developed a web‐based dashboard application that can be easily accessed by biology and clinical researchers for hypothesis generation and research verification.

## Results

### In‐depth acquisition of a quantitative human liver proteome

**Figure 1:**
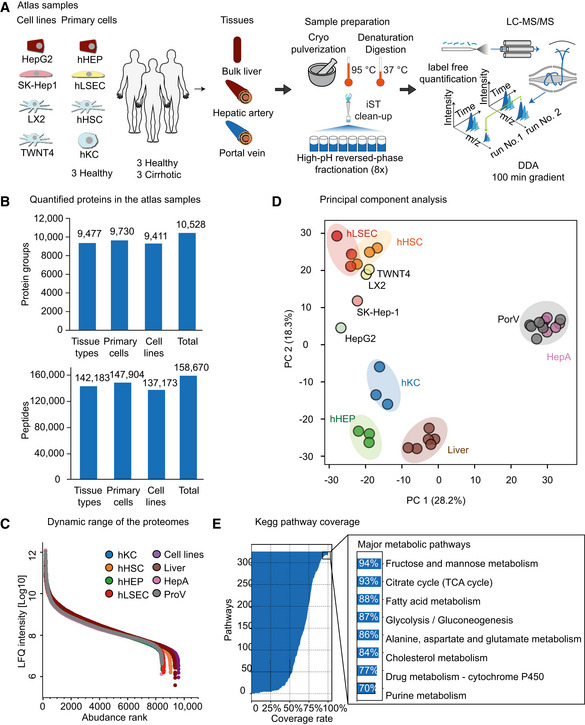
In‐depth characterization of the human liver proteome Overview of biological material used for generating the liver proteome atlas. (hHSC: hepatic stellate cell, hHEP: hepatocyte, hKC: Kupffer cell, hLSEC: liver sinusoidal endothelial cell, TWNT4 and LX2: immortalized human hepatic stellate cell line, SK‐Hep‐1: human hepatic adenocarcinoma cell line, HepG2: human liver cancer cell line). Number of biological replicates is *n* = 6 for bulk liver, hepatic artery and portal vein; *n* = 3 for hHEP, hLSEC, hHSC, hKC and *n* = 1 for HepG2, SK‐Hep1, LX2 and TWNT4. No additional replications of the experiment was done in laboratory.Total quantified proteome depth in tissues (*n* = 18), primary cells (*n* = 12), immortalized cell lines (*n* = 4) and all samples (*n* = 34). In all cases, *n* means biological replicates unless otherwise indicated. The upper and lower panel shows the number of quantified protein groups and peptides, respectively.Dynamic range of the different proteomes based on median intensity of label‐free quantification (LFQ) ordered by abundance rank (Liver: bulk liver biopsy, HepA: hepatic artery, PorV: portal vein, Cell lines: mixture of human liver‐derived immortalized cell lines). Number of biological replicates is same as Panel (A).Principal component analysis (PCA) of all proteomes based on their proteome profiles. For abbreviations, please refer to Panel (A).KEGG pathway coverage in this dataset, with major metabolic pathways highlighted.

To obtain a broad and representative overview of the liver proteome, we set out to measure diverse liver cell lines, primary cells, and human biopsies in great depth using the most advanced MS‐based proteomics workflows. We started with four commonly used immortalized cell lines, representing three hepatic cell types (Hep G2 for hepatocytes, LX2 and TWNT4 for hepatic stellate cells and SK‐Hep‐1, a human hepatic adenocarcinoma cell line). Next, we obtained primary hepatocytes (hHEPs), Kupffer cells (hKCs), liver sinusoidal endothelial cells (hLSECs), and hepatic stellate cells (hHSCs) isolated from three donors aged 57–64. Two donors had no signs of steatosis/fibrosis in the liver, and one had minimal portal inflammation (Table EV1). Characteristics and purity of the isolated cells were assessed by the provider—Samsara Sciences (Appendix). The methods they used for cell isolation have previously been shown to yield cells of high purity (DeLeve *et al*, 2004; Lecluyse and Alexandre, 2010). Finally, we analyzed biopsies of liver tissue, hepatic artery, and portal vein from six individuals undergoing liver transplantation (three healthy donors and three patients with liver cirrhosis, aged 39–59 years). From this total of 34 samples, we extracted peptides for MS analysis followed by eight‐fold fractionation using high‐pH reversed phase chromatography. We took care to completely disrupt the tissue, for adequate coverage of the extracellular matrix, and met the challenge of limited sample amount by employing a “loss‐less” nano‐fractionator (Kulak *et al*, 2017). In this part, our aim was to build a reliable proteome atlas and we therefore analyzed each fraction by liquid chromatography–tandem mass spectrometry (LC‐MS/MS) with a data‐dependent acquisition (DDA) method for an in‐depth proteome characterization (Fig 1A).

The resulting 272 raw data files from the 34 proteomes were analyzed using the MaxQuant software including label‐free quantification (LFQ) with the MaxLFQ algorithm, in which we require at least two peptides with minimum seven amino acids (Cox & Mann, 2008; Cox *et al*, 2014). Applying a stringent 1% peptide and protein false discovery rates (FDR), we identified and quantified 158,670 sequence unique peptides that were assigned to 10,528 protein groups (Fig 1B). These distinct groups sometimes resolved protein isoforms, mapping to 9,873 protein‐coding genes in the human genome. Our workflow quantified 9,477 proteins in all tissue types, 9,730 in all primary cell types and 9,411 in all cell lines. Thus, this most comprehensive human liver proteome dataset provides an excellent basis for systems‐wide analyses (Fig 1B and Dataset EV1).

**Figure EV1:**
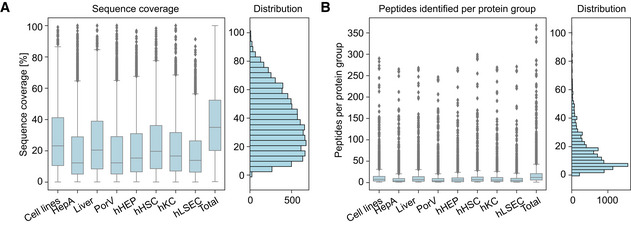
Proteomics data overview Box plots of sequence coverage distribution of all proteins quantified in each tissue/cell type, and in all samples combined (hHSC: hepatic stellate cell, hHEP: hepatocyte, hKC: Kupffer cell, hLSEC: liver sinusoidal endothelial cell, liver: bulk liver biopsy, HepA: hepatic artery, PorV: portal vein, Cell lines: mixture of human liver‐derived immortalized cell lines). Number of independent biological replicates is *n* = 4 for cell lines; *n* = 6 for HepA, Liver and PorV; and *n* = 3 for hHEP, hHSC, hKC and hLSEC. The gray line in the middle of the box is the median, the top and the bottom of the box represent the upper and lower quartile values of the data and the whiskers represent the upper and lower limits for considering outliers (Q3+1.5*IQR, Q1−1.5*IQR) where IQR is the interquartile range (Q3–Q1).Box plots of number of identified peptides per protein group in each tissue/cell type, and in all samples combined. Abbreviations and number of independent biological replicates are the same as Panel (A). The gray line in the middle of the box is the median, the top and the bottom of the box represent the upper and lower quartile values of the data and the whiskers represent the upper and lower limits for considering outliers (Q3+1.5*IQR, Q1−1.5*IQR) where IQR is the interquartile range (Q3–Q1).

The abundance of all quantified proteins spans more than six orders of magnitude (Fig 1C). Analyzing the data of our proteomic effort revealed a 35% median, aggregated sequence coverage (more than two million amino acids of the liver proteome), a very high value for shotgun proteomics (Fig EV1A). The median peptide number per liver protein was 12 but ranged up to 367 for Microtubule‐actin cross‐linking factor 1 (MACF1, Fig EV1B).

Principal component analysis (PCA) revealed distinct proteomes between tissue types, primary liver cell types, and the cell lines, with the first component capturing 28.2% of the variance setting apart hepatic arteries and portal veins from the rest (Fig 1D). The second component further separated hHEPs, hKCs, and liver tissue from hHSCs, hLSECs, and the cell lines, with 18.3% variance explained. Hepatocytes constitute about 80% of the liver cell population, therefore, should be characteristic of the bulk liver proteome. Thus, hepatocytes and biopsies of the liver tissue clustered very closely, whereas hHSCs and hLSECs exhibited higher similarity to each other. LX2 and TWNT4, the two immortalized hepatic stellate cell lines grouped closely with the corresponding primary cells, whereas HepG2 was more alike to the other cell lines than primary hepatocytes, indicating proteome divergence from the *in vivo* states and acquisition of common cell line features.

To investigate proteomic coverage of characteristic biological pathways of the liver, we extracted the 326 pathways of the KEGG pathway database (Kanehisa & Goto, 2000; Kanehisa *et al*, 2017). For fructose and mannose metabolism, citrate cycle, fatty acid metabolism, glycolysis/gluconeogenesis, and cholesterol metabolism, we obtained coverage between 84–94%, indicating that our data quantifies these processes nearly completely at the proteome level (Fig 1E). Conversely, pathways that are not expected to have biological functions in the liver, say nicotine addiction and the olfactory transduction pathways, had less than 4% pathway coverage.

### Distinct proteome features between liver parenchyma and blood vessels

**Figure 2:**
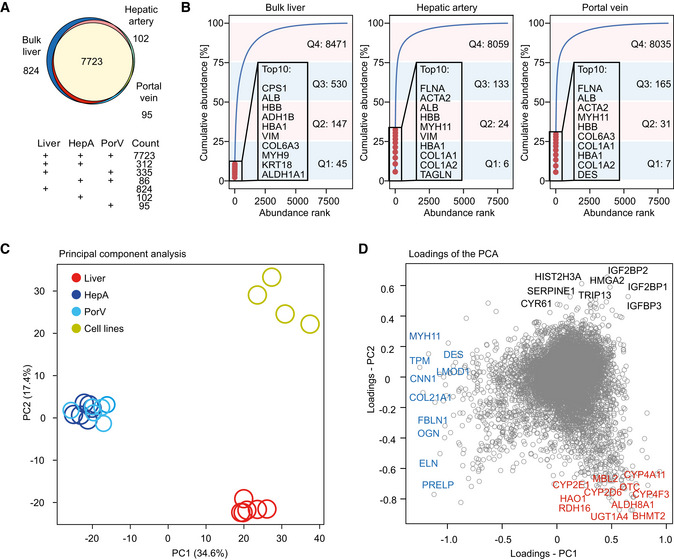
Comparative analysis of the liver tissue proteomes Commonly and exclusively quantified proteins in bulk liver tissue, hepatic artery, and portal vein.Cumulative protein abundance of liver biopsy, haptic artery, and portal vein as a function of protein rank, with the total of the top 10 abundant proteins and number of proteins that comprise four quartiles indicated.PCA of liver, hepatic artery, portal vein, and cell lines.Loadings of the PCA in Panel (C) with proteins that contribute most to the variance for the three clusters annotated.

To capture the proteome differences between liver parenchyma and extrahepatic blood vessels, we characterized biopsies of the liver tissue, portal vein, and hepatic artery. In total, 9,477 proteins were quantified across different tissues, of which 81% were common to all tissues. We exclusively quantified 824 proteins in liver biopsy but only 86 exclusive to the two blood vessels (Fig 2A), indicating both higher proteomic complexity of the liver biopsies, and the presence of blood vessels in the portal triad. The 86 proteins are characteristic of the unique muscle fiber structure of blood vessels, such as Myosin‐7B, Tropomyosin alpha‐1 chain, and Plectin. They tend to be present in moderate‐to‐low abundance and most of them are typically not covered by standard single‐run tissue proteomics workflows. To illustrate, there are only 12 unique proteins to HepA or PorV among the 3,000 most abundant proteins, and 24 among the top 5,000. When extracting the most abundant proteins in liver biopsy based on intensity of LFQ, we found metabolic enzymes (carbamoyl‐phosphate synthase 1 (CPS1), which is heavily downregulated in NAFLD (De Chiara *et al*, 2018), alcohol dehydrogenase 1B (ADH1B), and retinaldehyde dehydrogenase 1 (ALDH1A1)), blood proteins (albumin (ALB) and hemoglobin subunits (HBA1, HBB)) and structural constituents of the cytoskeleton (vimentin (VIM), type VI collagen (COL6A3), myosin (MYH9), and keratin 18 (KRT18); Fig 2B). In contrast, the most abundant hepatic artery proteins were almost exclusively cytoskeleton proteins with filamin‐A (FLNA), smooth muscle alpha‐2 actin (ACTA2), myosin (MYH11), vimentin (VIM), type I collagen (COL1A1, COL1A2), and transgelin (TAGLN) apart from albumin and hemoglobin subunits among the top 10 (Fig 2B). Analysis of the top abundant proteins in liver biopsy and blood vessels revealed their structural and functional characteristics. The high abundance of blood‐related proteins (ALB, HBB, and HBA1) could be due to some residual blood contamination, although the samples used in the analysis were flushed and rinsed upon sampling.

Of note, we found that half of the total measurable proteome mass in liver is comprised of less than 200 proteins. Adding the next 523 proteins accounts for 75% of the total measurable proteome mass, whereas the remaining 25% is composed of an astonishing 8,471 proteins. The top abundant protein in liver tissue—CPS1, has a quantitative signal of more than 1 million‐fold higher than the least abundant protein (ER lumen protein‐retaining receptor 3, KDELR3) that we quantified. This also explains why liver is a very challenging tissue in proteomic analysis. This uneven distribution is even more pronounced in the hepatic artery and portal vein, with half of the proteome mass composed of merely 30 and 38 proteins, respectively.

Next, we investigated proteins that contribute the most to the separation of proteomes between liver biopsy and the blood vessels. In PCA, as already mentioned, biological replicates of liver biopsies and those of blood vessels clustered closely together (Fig 2C). Proteins that are characteristic of the blood vessels such as cytoskeleton, and extracellular matrix proteins drive the first dimension of this separation, together with metabolic enzymes in the bulk liver tissue such as members of the cytochrome P450 superfamily: CYP2E1, CYP4A11, CYP4F3, CYP2D6, and enzymes in alcohol metabolism: ALDH8A1, RDH16, HAO1. When adding hepatic cell lines in this analysis, we found proteins involved in the regulation of cell differentiation and proliferation, such as CYR61, SERPINE1, HIST2H3A, RORC, IGF2BP1, HMGA2; regulatory proteins in cell cycle: HMGA2 and TRIP13 and proteins involved in chromatin organization: HIST2H3A, JMJD6, HMGA2 (Fig 2D). These examples illustrate how our resource can connect quantitative protein expression with functional roles of tissues and cell lines.

### Proteome differences between primary cell types reflect their functional roles

**Figure 3:**
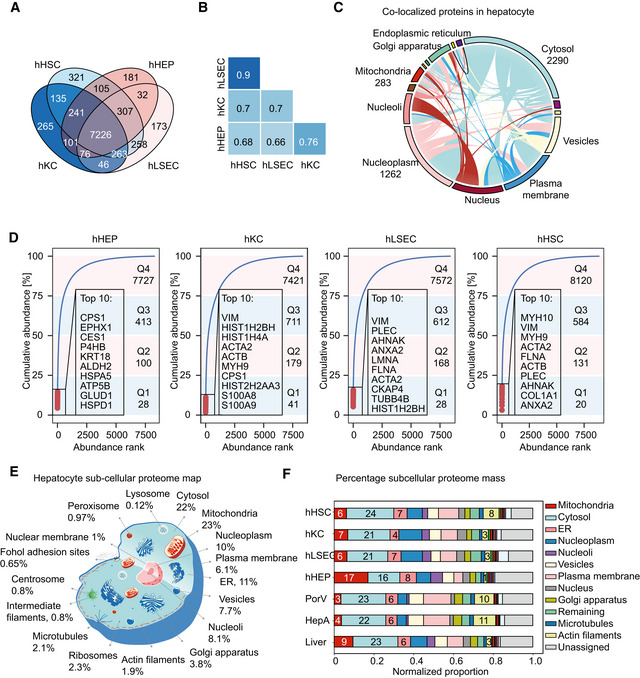
Comparative analysis of liver cell type proteomes Commonly and exclusively quantified proteins in liver cell types (hHSC: hepatic stellate cell, hHEP: hepatocyte, hKC: Kupffer cell, hLSEC: liver sinusoidal endothelial cell).Pair‐wise correlation of the proteomes of the four primary cell types, with Pearson correlation coefficients noted.Circos plot representing proteins predicated to be co‐localized in subcellular compartments.Cumulative protein abundance of liver cell types as a function of protein rank, indicating the total of the top 10 abundant proteins and number of proteins that comprise the four quartiles.Schematic representation of the subcellular mass composition of an average hepatocyte.The bar plot shows the contribution of each organelle to total cellular protein mass, also accounting for unassigned proteins. Percentage of cytosol, mitochondria, nucleoli, plasma membrane, and actin filaments is indicated.

Among the non‐parenchymal cells populating the liver, LSECs, hHSCs, and hKCs predominate. We quantified more than 8,200 proteins in primary cells of hepatocytes and each of these cell types, and more than 7,200 across all of them (Fig 3A). Among the proteins that were uniquely identified in one cell type, 284 were detected in at least two of three biological replicates (Fisher exact test *P* < 0.05, one cell type against the rest), making them candidates for cell type‐specific proteins. As expected by their very different functions, proteome similarities were modest (average Person correlation coefficient of abundance was 0.73). The one exception was between hLSECs and hHSCs with a correlation coefficient of 0.9 (Fig 3B). The high proteome similarity between hLSECs and hHSCs in the liver has been previously observed in both proteomics and single‐cell transcriptomics studies (Aizarani *et al*, 2019; Ölander *et al*, 2020). The cumulative protein abundance showed an uneven distribution similar to the bulk liver tissue.

Interestingly, 25% of total protein mass in hepatocytes is comprised of only 28 most abundant proteins (Fig 3D). The top 10 are primarily metabolic enzymes including CPS1, liver carboxylesterase 1 (CES1), protein disulphide‐isomerase (P4HB), aldehyde dehydrogenase (ALDH2), ATP synthase subunit beta (ATP5B), and glutamate dehydrogenase 1 (GLUD1). The only exceptions were heat shock proteins A5 and D1 and keratin 18, a widely used marker of hepatocyte cell death. In Kupffer cells, cytoskeleton components such as vimentin, actin isoforms, and myosin are among the top abundant proteins. In the top 10 most abundant proteins, they have S100A8 and S100A9, who play a prominent role in the regulation of inflammatory processes and immune response (Pruenster *et al*, 2016). Their heterodimer, termed calprotectin, is also highly expressed in neutrophils and monocytes. The nature of the most abundant proteins reflects their functional roles such as migration along the sinusoids and for the immune response when encountering infections. In both hLSEC and hHSC, again unlike in the hHEPs, the top 10 abundant proteins are mostly cytoskeletal framework components, in this case presumably required for forming the endothelial fenestrae by hLSEC and for the maintenance of morphology and cellular adhesion of hHSC (Fig 3D).

**Figure EV2:**
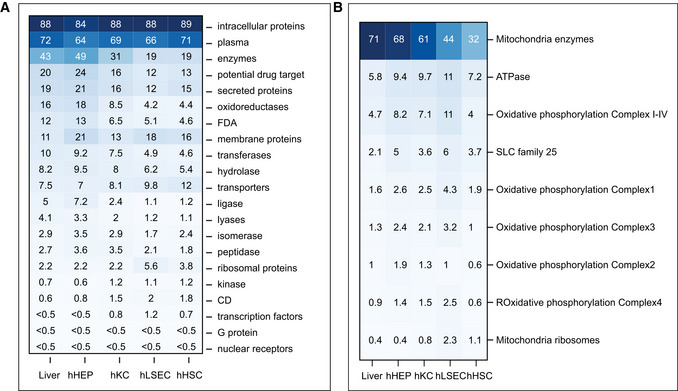
Cellular mass composition based on protein classes Percent protein mass by protein classes. Protein class annotation was based on the HPA classification.Percent protein mass in the mitochondria by selected protein classes. Oxidative phosphorylation is the sum of Complex 1–4.

The same proteins can be present in different cell types in very different amounts, depending on their functional demands. To investigate the cellular mass composition at the protein class level, we followed the annotation of proteins into 21 classes, such as enzymes, secreted protein, and drug targets, of the Human Protein Atlas (HPA; Uhlen *et al*, 2016). This revealed major differences between the cells, for example, 21% of the protein mass of primary hepatocytes are classified as secreted proteins, as compared to only 12–16% in the other cell types (Fig EV2A). This is expected since a role of hepatocytes is to produce and secrete proteins into the blood. Our data show that enzymes together comprise as much as 49% of total proteome mass in hepatocytes. The number drops to 31% in hKC, and 19% in both hLSEC and hHSC, whereas hLSEC and hHSC express higher levels of transporters, cluster of differentiation (CD) markers and ribosomal proteins compared to hHEP and hKC. In addition, hLSEC features nearly four times higher abundance of transcription factors as hHEP. Hepatocytes also have the highest abundance of FDA‐approved drug target proteins (13%) and potential drug targets (24%), reflecting a focus on modulating metabolic liver functions and underlining the importance of liver disease as comorbidity (Fig EV2A). A potential use of our resource is to understand the quantitative distribution of drug targets under consideration and to highlight proteins or regulatory pathways present in cell types of therapeutic interest.

To investigate the proteome composition of subcellular compartments in the liver, we predicted subcellular proteome maps by mapping the quantified proteins to 32 subcellular localizations according to Gene Ontology Cellular Component (GOCC). According to this analysis, mitochondria comprise 23% of total proteome mass in hepatocytes, followed by the cytosol and the endoplasmic reticulum, with 22 and 11%, respectively (Fig 3E, Dataset EV2). As many proteins co‐localize to more than one organelle (Fig 3C), we normalized the proteome proportions based on the GOCC information. This revealed that mitochondria have three times the total proteome mass in hepatocytes as in other cell types (Fig 3F, Dataset EV2). Conversely, hepatocytes have the least contribution to proteome mass from the cytoskeleton illustrated by seven times lower levels of actin filaments than in hHSCs and one thirds of those of hKCs and hLSECs (Fig 3F).

To further dissect proteome composition of mitochondria in hHEPs, we determined protein components of total mitochondrial enzymes (68%), oxidative phosphorylation (OXPHOS) complex I–IV (8.2%), ATPase (9.4%), solute carriers family 25 (5%), and mitochondria ribosomes (0.4%). These proportions were quite different in the other cell types (Fig EV2B). As an example, the summed proportion of OXPHOS complex I–IV in hepatocyte mitochondria is twice of that in hHSCs. Among the 195 solute carrier proteins quantified in our atlas data, about 15% belong to the mitochondrial carrier transporter family 25 (SLC25), accounting for up to 6% of the mitochondrial mass. SLC25 is the largest solute transporter family in humans and central to mitochondrial function (Perland & Fredriksson, 2017; Ruprecht & Kunji, 2020). Among all 66 SLC families, SLC25 accounted for an astonishing 70% of total solute carrier proteins in hepatocytes, and 45–57% in the other cell types.

### Proteome ruler and cell type‐specific proteins

**Figure EV3:**
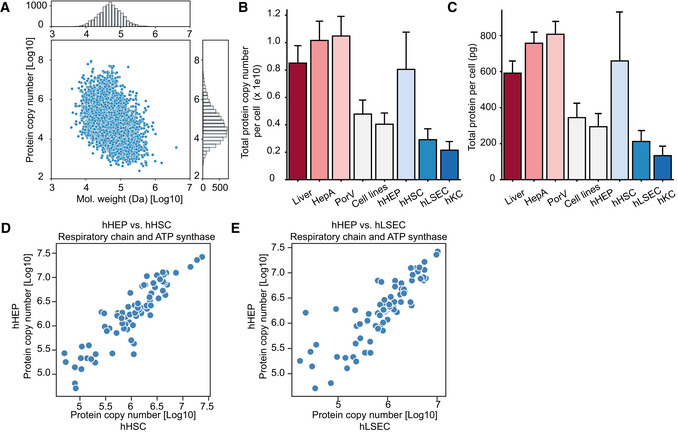
Protein copy number estimation AAveraged protein copy number versus molecular weight in the hepatocyte proteome, with histogram on the *x*‐ and *y*‐axis representing distribution of the molecular weights and protein copy numbers, respectively. Averaged protein copy numbers are derived from *n* = 3 independent biological samples.B, CTotal protein copy number (B) and total protein mass per cell (C) in liver tissues and cell types estimated by the “proteome ruler” approach. Number of biological replicates is *n* = 6 for Liver, HepA, PorV; *n* = 4 for Cell lines and *n* = 3 for hHEP, hHSC, hLSEC and hKC. Values are presented as mean ± s.d.D, EProtein copy number for members of the oxidative phosphorylation complex I–V in hepatocytes and hepatic stellate cells (D), sinusoidal endothelial cells (E).

**Figure 4:**
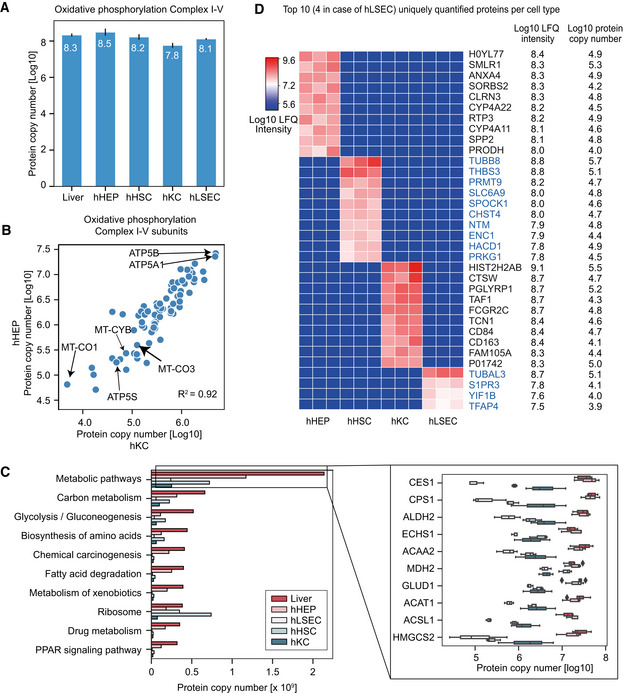
Quantitative analysis of sub‐cellular proteomes and biological pathways Total protein copy number of the oxidative phosphorylation complex I–V in bulk liver tissue (*n* = 6 independent biological replicates) and primary cell types (isolated primary cells from *n* = 3 individuals for each cell type). Values are presented as mean ± s.d.Protein copy number for members of the oxidative phosphorylation complex I–V in hepatocytes and Kupffer cells with Pearson correlation coefficient indicated.Protein copy number estimation of the KEGG pathways. The left panel shows the estimated total number of proteins per cell of top 10 most abundant pathways in liver biopsy in terms of total molecules of proteins associated. The right panel shows the protein copy numbers of top 10 most abundant proteins in liver biopsy that are associated with metabolic pathways and their distribution in liver biopsy (*n* = 6), hHEP (*n* = 3), hLSEC (*n* = 3), hHSC (*n* = 3), and hKC (*n* = 3). The black line in the middle of the box is the median, the top and the bottom of the box represent the upper and lower quartile values of the data and the whiskers represent the upper and lower limits for considering outliers (Q3+1.5*IQR, Q1−1.5*IQR) where IQR is the interquartile range (Q3–Q1).Top 10 uniquely quantified proteins per cell type with label‐free quantification (LFQ) intensities [Log_10_] and copy numbers [Log_10_].

Given the pronounced differences in protein abundance across cell types at the subcellular organelle and protein class level, we were interested in estimating absolute copy numbers for all proteins. We applied the “proteomic ruler” approach that uses the total histone mass to cellular DNA (Wisniewski *et al*, 2014). This concept usually assumes diploidity, whereas hepatocytes reportedly have between one and four nuclei (Thoma, 2018); therefore, our copy numbers are likely to be underestimated (Fig EV3A). Our calculations resulted in two to eight billion protein molecules, corresponding to 150–700 pg of protein mass per cell across different cell types (Fig EV3B and C). Our rough estimation allows us to infer for instance, the stoichiometric ratios of respiratory chain subunits of associated protein complexes and ATP synthase. This shows that there are approximately 128 million protein molecules of the ATP synthase in hepatocytes, with the OXPHOS complex I–IV having 15–77 million protein copies totaling 305 million in the OXPHOS complexes I–V, up to four‐fold higher than those in the other cell types (Fig 4A). Protein subunits belonging to the same complex can be very different, illustrated by the more than 100‐fold difference between the most and least abundant ATP Synthase subunits (ATP5B to ATP5S; Fig 4B), reflecting the regulatory role of the latter as a coupling factor (Belogrudov & Hatefi, 2002; Jonckheere *et al*, 2012). Subunits alpha (ATP5A1) and beta (ATP5B) forming the catalytic core in the F1 domain of the ATP Synthase, however, have very similar copy numbers with a ratio between 0.8–0.97 (ATP5A1: ATP5B; Fig 4B) for the four cell types, in excellent agreement with their assembly stoichiometry of 1:1 (Walker *et al*, 1985; He *et al*, 2018). The F0 membrane domain is an assembly of single‐copy subunits and we found similar copy numbers between them (within two‐fold difference from the mean). The only exceptions were the mitochondrial DNA‐encoded ATP6 and ATP8, with less than three‐fold of the mean copy number, possibly reflecting differences in protein synthesis by cytosolic‐ and mitochondrial ribosomes (Table EV2). Similarly, MT‐CO1 and MT‐CO3, two of the three mitochondrial DNA‐encoded subunits in the cytochrome c oxidase complex (Complex IV), also had the lowest number of protein copies compared to the rest (ranking 12^th^ and 14^th^ among the 14 subunits). The same applies to MT‐CYB in Complex III. Protein complexes consist of subunits that are produced in excess. Thus, our data may point to a differential role of mitochondrial and cytosolic protein biogenesis for the OXPHOS complexes. Even at an approximate level, our estimates allow modeling the stoichiometric ratio of the overall respiratory chain machinery (Complex I–V) between different cell types based on all subunits. The data indicated that approximately 300 million protein molecules in these complexes were in hepatocytes compared to only 55 million in Kupffer cells (Fig 4A).

Taking the copy number concept to another level, we extracted them for entire KEGG pathways. For instance, about 1.2 billion protein molecules in hepatocytes carry out metabolic pathway functions, whereas 240–720 million do so in the other liver cell types (Fig 4C). The top abundant metabolic enzymes—Liver carboxylesterase 1 (CES1) and Carbamoyl‐phosphate synthase (CPS1)—alone have more than 40 million protein copies each (Fig 4C).

Several genes containing non‐synonymous single nucleotide polymorphisms (SNPs) have been identified to contribute to the pathogenesis of NAFLD, including PNPLA3, MBOAT7, GCKR, and HSD17B13 (Trépo & Valenti, 2020). Our copy number catalogue indicated that hHEPs express the highest of each of these proteins with the only exception of MBOAT7, which is most abundant in hHSCs (25‐fold as that in hHEPs). MBOAT7 is a membrane‐bound, lysophopholipid acyltransferase. Its rs641738C>T allele has been reported to be associated with fibrosis in a number of liver diseases, and it was recently shown that a loss of MBOAT7 leads to liver fibrosis, to which the mechanism is incompletely understood (Thangapandi *et al*, 2021). Our data show that MBOAT7 has the highest expression in hHSCs, which might point to new directions in elucidating this mechanism given the prominent role of hHSCs in fibrogenesis. PNPLA3 is a lipid droplet‐associated protein with hydrolase activity towards triglycerides. Individuals carrying an I148 allele on PNPLA3 have a two‐fold increased risk for developing NAFLD (Romeo *et al*, 2008; Speliotes *et al*, 2010). We quantified it in only hHEPs and hHSCs with approximately 21,000 and 4,700 copies per cell, respectively. This underlines again the importance of non‐parenchymal cells in development of steatosis or NASH.

TGF‐beta receptor and PDGF are required for hHSC activation and inhibiting these pathways are under active investigation in terms of their anti‐fibrotic potential. We further looked into their abundance levels and found that indeed, hHSCs have the highest levels of PDGF‐alpha and beta (PDGFRA, PDGFRB) as well as TGF‐beta receptor type 1 and type 2 (TGFBR1, TGFBR2) among all cell types, with copy numbers ranging from 150,000 to 2,000,000 (Table EV3). Although copy numbers in the other cell types are seven‐fold lower on average, their higher proportion in the liver adds up to similar copy numbers. In this way, our resource provides useful information for therapeutically relevant proteins across liver cell types in relation to potential toxic effects. This builds a case why cell type‐specific targeting is more effective than global approaches with less adverse effects (Klein *et al*, 2012, 2019).

Next, we investigated unique protein expression between cell types. There were only 109 proteins uniquely quantified in all the biological replicates of each cell type, and we define them as “cell‐type specific” (Table EV4). Among the top 34 unique proteins per cell type, about 21 are among the top 5,000 abundant proteins within corresponding cell types, making it unlikely that they are uniquely detected in these cells for technological reasons (Fig 4D). Our data confirm known markers, for instance, CD163, a macrophage scavenger receptor, as a known marker of macrophages that is commonly used for Kupffer cell isolation. Three other proteins in the Kupffer cell‐specific protein panel, namely peptidoglycan recognition protein 1 (PGLYRP1), cathepsin W (CTSW), and CD84 are involved in the immune response. Apart from the liver, these proteins also have “enriched expression” according to RNA‐seq data in the HPA in bone marrow and lymph nodes, in agreement with the similarity of Kupffer cells as resident macrophages with the infiltrating macrophages from the bone marrow before they migrate to the liver. Among the top 10 hHEP‐specific proteins, six have “enriched expression” in the liver, namely small leucine rich protein 1 (SMLR1), clarin 3 (CLRN3), members of the cytochrome P450 family (CYP4A22, CYP4A11), receptor transporter protein 3 (RTP3), and secreted phosphoprotein 2 (SPP2) with protein copy numbers up to 180,000 (Table EV4). RTP3 and SPP2 are “exclusively expressed” (meaning exclusively detected) in the liver according to the HPA to which our data assigns quantitative and cell type resolved information, with more than 70,000 and 50,000 protein copies per cell, respectively (Fig 4D). A recent study has identified CLRN3, as a cell surface protein for hepatocyte‐like cells derived from induced pluripotent stem cells (Mallanna *et al*, 2016). Interestingly, the top hHEP‐specific protein is an uncharacterized protein (H0YL77). Apart from hepatocytes, it was also quantified in the bulk liver samples and the HepG2 cell line, demonstrating its specificity to hepatocytes. When calculating the Pearson correlation coefficients of its abundance profile with other proteins, the highest correlation was to mitochondrial fission factor (MFF; *r* = 0.99; Table EV5).

Among the top 10 hHSC‐specific proteins, the HPA assigns three as primarily expressed in the cerebellum or cerebral cortex, namely testican‐1 (SPOCK1), neurotrimin (NTM), and ectodermal‐neural cortex 1 (ENC1). It has been hypothesized that HSCs derived from the neural crest due to their similar gene expression pattern to that of neural cell types (Sato *et al*, 2003), which our data support at the quantitative level for these uniquely expressed proteins. Note that the neural crest origin of HSC has been challenged, and another hypothesis suggests multipotent mesenchymal progenitor cells as the origin, particularly for these cells also give rise to neural cells and mesenchymal lineages (Friedman, 2008; Hellerbrand, 2013). In line with the fundamental role of HSCs in producing extracellular matrix components and the initiation, progression, and regression of liver fibrosis (Mederacke *et al*, 2013), we found carbohydrate sulfotransferase 4 (CHST4) among their top 10 specific proteins. This protein plays an important role in lymphocyte homing at sites of inflammation. It has been linked to liver disease and was shown to predict the prognosis of hepatocellular carcinoma (Zhang *et al*, 2020; Hu *et al*, 2021). Only four proteins were hLSEC‐specific by our criteria, likely due to their proteome similarity with hHSC. The four proteins are tubulin alpha chain‐like 3 (TUBAL3), transcription factor AP4 (TFAP4), Yip1 interacting factor homolog B (YIF1B) and sphingosine‐1‐phosphate receptor 3 (S1PR3; Fig 4D). Together, our analysis identifies known as well as so far undescribed cell type‐specific proteins in the liver and provides their abundance levels.

### Functional specialization of human liver cell types

**Figure 5:**
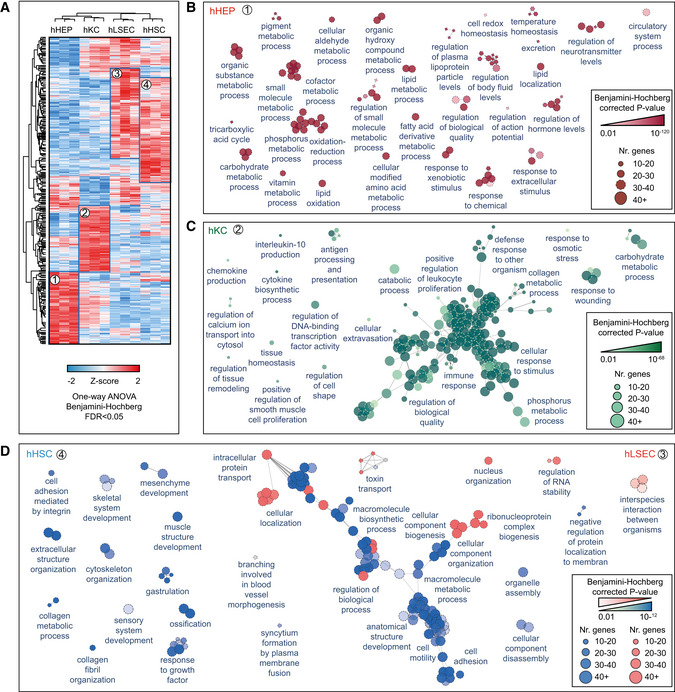
Liver cell type functional specialization map Unsupervised hierarchical clustering of proteins significantly differentially abundant across cell types, with columns showing three biological replicates (*n* = 3) of four cell types and rows significant proteins. Significance was calculated by one‐way ANOVA followed by Benjamini–Hochberg correction for multiple hypothesis testing (FDR < 0.05). Frames and numbers indicate four clusters of proteins highly enriched in each cell type.GOBP map specific for hHEP (proteins in cluster 1). Each circle represents an individual biological process term significantly enriched, with the number of proteins and FDR‐corrected *P*‐value indicated by size and degree of transparency. Significance was calculated by Fisher’s exact test followed by Benjamini–Hochberg correction for multiple hypothesis testing (FDR < 0.01). The leading term given by the most proteins associated within a group is indicated.GOBP map specific for hKC (proteins in cluster 2). Significance was same as Panel (B).Comparative analysis of GOBP enrichment for hHSC and hLSEC from Cluster 3 and Cluster 4 from Panel (A), with blue terms specific for hHSC and pink specific for hLSEC and gray terms shared between them. Significance was same as Panel (B).

**Figure EV4:**
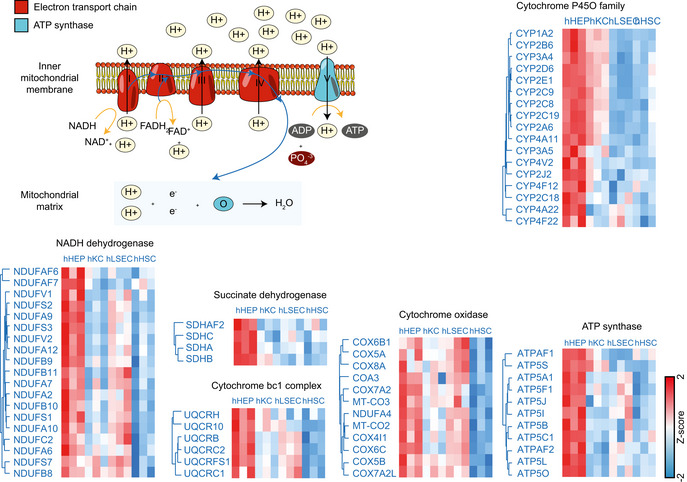
Hepatocyte‐enriched proteins in respiratory chain and drug metabolism Clustering of proteins significantly differentially abundant across the four cell types that belong to the electron transport chain and ATP synthase. Values are presented as mean protein abundance followed by *Z*‐score normalization across cell types (*n* = 3 independent biological replicates for each cell type).

We further investigated the functional characteristics of the different liver cell types in an unbiased manner using ANOVA, which showed that nearly half of the liver proteome was significantly different in at least one of them (4,173 proteins, Methods, Dataset EV3). After hierarchical clustering based on Euclidean distance, four large clusters of cell type characteristic proteins appeared (1,000, 900, 1,168 and 1,331 proteins enriched in hHEPs, hKCs, hLSECs, and hHSCs, respectively, Fig 5A). These panels are very distinct between hHEPs and hKCs but overlap between hLSECs and hHSCs. Reflecting its active role in energy metabolism and maintaining homeostasis, the corresponding GO terms are highly and significantly enriched in hHEPs, along with complement activation, blood coagulation, and regulation of blood lipoprotein levels (Fig 5B). Higher protein levels of the oxidative phosphorylation machinery in hepatocytes from the above‐targeted analysis was also reflected in this unbiased analysis (Fig EV4).

As expected, GO terms associated with immune responses were highly enriched in hKCs, including antigen presentation and processing, cytokine and chemokine production, as well as cell motility (Fig 5C), possibly required for movement along the liver sinusoids (MacPhee *et al*, 1992). Surprisingly, several metabolic pathways were also significantly enriched, including phosphorus metabolic process, monosaccharide metabolic process and collagen metabolic process. This is demonstrated by a higher abundance of dehydrogenases, for example, GPD1L (glycerol‐3‐phosphate dehydrogenase 1‐like protein), GAPDH (glyceraldehyde‐3‐phosphate dehydrogenase), ME1 (NADP‐dependent malic enzyme); carbohydrate kinases: PGK1 (phosphoglycerate kinase 1), PFKL (ATP‐dependent 6‐phosphofructokinase) as well as ALDOA, a fructose‐bisphosphate aldolase known to be predominantly expressed in skeletal muscle.

**Figure EV5:**
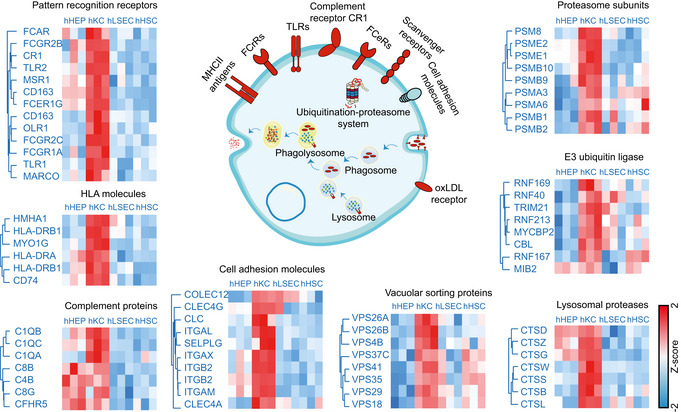
Kupffer cell‐enriched proteins in antigen processing and presentation pathway Clustering of proteins significantly differentially abundant across the four cell types that are related to the immune response and protein degradation. Values are presented as mean protein abundance followed by *Z*‐score normalization across cell types (*n* = 3 independent biological replicates for each cell type).

Proteases, such as cathepsins (CTSG, CTSB, CTSD, CTSH, CTSL, CTSS, CTSW, and CTSZ) which are proteolytic enzymes that contribute to pathogen killing in lysosomes (Pires *et al*, 2016), proteins of the core machinery of ubiqutination and proteasomal degradation and matrix metalloproteinases (MMP8, MMP9, MMP25) that have crucial regulatory functions have their highest abundance in Kupffer cells. Further reflecting the roles in engulfing pathogens into lysosomal compartments to undergo degradation pathways, vacuolar protein sorting‐associated proteins involved in cargo transport, such as VPS11 and VPS26A, were most highly expressed compared to the other cell types (Fig EV5).

As most hHSCs‐ and hLSECs‐enriched proteins overlapped, we did a comparative functional enrichment analysis on proteins from cluster three and four in Fig 5A. Enriched terms were termed dominant in a cell type if the percentage of associated proteins from the cell type is larger than the other. Among the 26 terms specific for hLSECs, a considerable proportion relates to ribonucleoprotein complex biogenesis, gene expression and RNA localization. Hence, levels of 40S‐ and 60S‐ ribosomal subunits as well as 39S‐ and 28S‐ mitochondrial ribosomal proteins, members of the nuclear pore complex, RNA helicase, and translation initiation among the others where comparatively higher, reflecting higher biosynthetic activity due to the important role of LSEC in liver homeostasis, especially since LSEC is the first barrier of nutrients and “pathobionts” entering the human body through the gut.

**Figure EV6:**
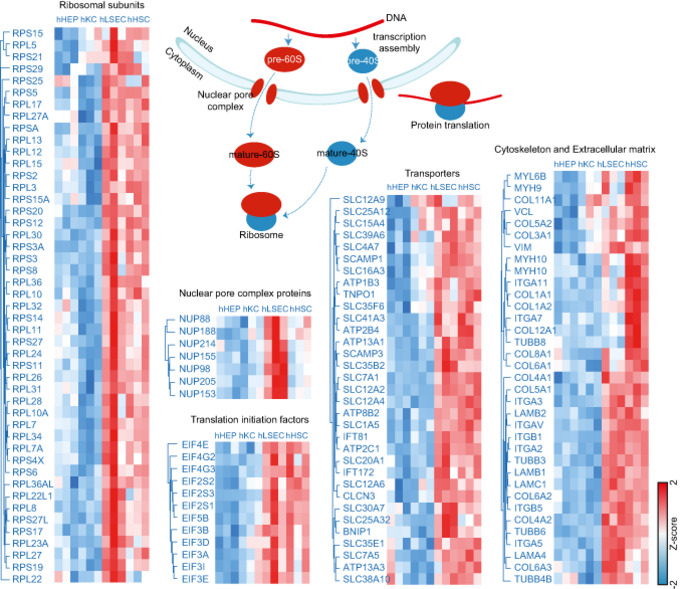
LSEC‐enriched proteins in ribosomal biogenesis and translation and HSC‐enriched proteins in cytoskeleton and extracellular matrix Clustering of proteins significantly differentially abundant across the four cell types that are involved in ribosomal biogenesis and translation, as well as cytoskeleton and extracellular matrix. Values are presented as mean protein abundance followed by *Z*‐score normalization across cell types (*n* = 3 independent biological replicates for each cell type).

LSEC constitutes the sinusoidal fenestrae, and plays an essential role in the exchange of solutes, metabolites, fluid, and particles between the hepatocytes and sinusoidal blood (Knolle & Wohlleber, 2016). Accordingly, proteins involved in intracellular transport, such as many SLC family members, were highly abundant compared to other cell types (Fig EV6). Membrane proteins with virus receptor activity involved in interspecies interaction were also highly enriched such as CD46, ICAM1, PVR (poliovirus receptor), EPHA2 (ephrin type‐A receptor 2, which acts as a HCV receptor in hepatocytes and facilities its entry), as well as ANPEP (also known as CD13), DPP4 (also known as CD26) which both have human coronavirus receptor activity (Peck *et al*, 2017; Sungnak *et al*, 2020; Tang *et al*, 2020), presumably rendering LSEC more sensitive or susceptible to viral infection or translocation through receptor‐mediated endocytosis or fusion. Among the HSC‐specific enriched terms, many relate to anatomical structure development and extracellular structure organization (Fig 5D). Highly abundant cytoskeleton component proteins such as actin and tubulin, motor protein myosin, membrane‐cytoskeletal protein vinculin, transmembrane receptor integrins and extracellular matrix proteins (laminin and collagens) all belong to this category. Alpha‐smooth muscle actin (α‐SMA) is a marker for HSC activation but its expression is not unique to HSC, with up to 10‐fold lower copy numbers in the other cell types in our resource.

### Primary cell culture reveals hepatic stellate cell activation‐related proteome signatures

Functional studies related to the liver and its cell types are mostly performed in primary or immortalized cell lines due to convenience and reproducibility. We previously compared immortalized murine hepatocyte cell lines to their cognate primary cells, which revealed rearrangement of characteristic metabolic processes, decrease in mitochondria function, while insulin signaling remained intact (Pan *et al*, 2009). In mouse tissue and isolated hepatocytes, expression of complement components gradually decreased as one aspect of extensive overall proteome remodeling during cell culture (Azimifar *et al*, 2014). In another proteome study, a decrease in expression of cytochrome P450 family was observed in human hepatocyte cell culture (Heslop *et al*, 2017).

**Figure 6:**
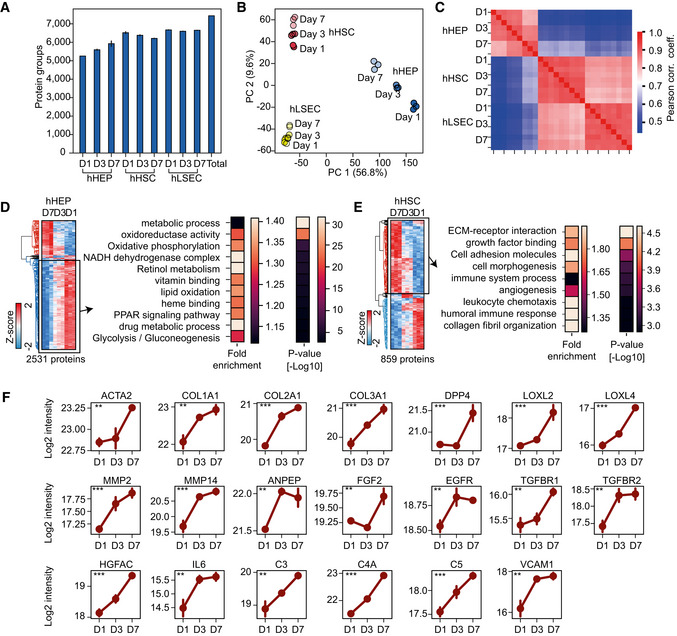
Proteomics analysis of human liver primary cells upon cell culture Total quantified proteins at day one, three and seven upon primary cell culture in hepatocytes (hHEP), hepatic stellate cells (hHSC) and liver sinusoidal endothelial cells (hLSEC). In all cases, number of biological replicates is 3. Values are presented as mean ± s.d. No additional replications of the experiment was done in laboratory.PCA showing proteome dynamics of primary cells upon cell culture.Pair‐wise Pearson correlation of proteomes during cell culture.Significantly changing proteins during primary hHEP cell culture, indicating significantly enriched GO terms on the cluster of down‐regulated proteins. Significance was calculated by Fisher’s exact test corrected for multiple hypothesis testing with FDR < 0.05.Significantly changing proteins during primary hHSC cell culture, indicating significantly enriched GO terms on the cluster of up‐regulated proteins. Significance was calculated by Fisher’s exact test corrected for multiple hypothesis testing with FDR < 0.05.Protein expression patterns over the course of primary hHSC culture (*n* = 3). Values are presented as mean ± s.d. Significance was calculated by ANOVA followed by Benjamini–Hochberg correction for multiple hypothesis testing (FDR < 0.05) with a significance level of **P < 0.01, and ***P < 0.001.

Our liver proteomics workflow could shed light on which cellular functions are retained in human liver cells during adaption from an *in vivo* like state to a cell line state. To test this hypothesis, we performed primary cell culture of hHEPs, hHSCs, and hLSECs and investigated their proteomic changes at day‐1, day‐3, and day‐7. We selected the three time points based on a previous study in which we observed substantial proteome remodeling during 7‐day culture of primary hepatocytes freshly isolated from mice (Azimifar *et al*, 2014). We did not further extend the culture period or sample more frequently due to limitations in sample availability and the limited growth potential of primary cells in culture. During the cell culture experiment, we chose optimal culturing conditions for each primary cell type according to the provider’s instructions and systematically controlled the viability and confluency to avoid introducing proteome changes due to sub‐optimal growth conditions (Methods). We acquired the mass spectra in data‐independent acquisition (DIA) mode with FAIMS, and *in silico* library (directDIA) was used for data processing (Bruderer *et al*, 2015; Methods). In biological triplicates and single‐run proteomic measurements, 7,501 proteins were quantified in total (Fig 6A, Dataset EV4). Hepatocytes had the largest proteome alterations comprising 41% of the total in hHEP (2,531 proteins, ANOVA, FDR < 0.05) followed by hHSC (13%) and hLSEC (3%; Dataset EV4). Despite this proteome drift, the same cell types still cluster in PCA, with the separation of hHEPs from hHSCs and hLSECs in the first component already explaining 57% of the variance (Fig 6B). This recapitulates the data from uncultured primary cells as described above, in which the proteomic profiles of hHSC and hLSEC are most alike. The PCA clearly shows the step‐wise proteome shift of these primary cells from day‐1 to day‐7, which was further reflected by decreased Pearson correlation coefficients (0.91 from day‐1 to day‐3 and 0.8 from day‐1 to day‐7 in case of hHEP; Fig 6C).

To understand the proteome shifts at the level of biological processes, we performed functional enrichment analysis of the differentially abundant proteins. For hHEP, this unveiled an upregulation of proteins involved in regulation of cell shape, adhesion, and migration, as exemplified by actin and actin‐binding proteins (myosin, tropomyosin, gelsolin, drebrin) as well as extracellular matrix proteins (VWF, tenascin‐X, HSPG2, collagen type III, IV and VI), presumably reflecting loss of the supporting *in vivo* structural framework as well as mechanical adaption to the *in vitro* environment. Conversely, metabolic or energy homeostatic processes were downregulated, accounting for half of the altered proteome, including more than 70 members of the oxidative phosphorylation machinery components. Likewise, we observe a downregulation of GO terms related to binding vitamins and metal, along with PPAR (peroxisome proliferator‐activated receptor) signaling. PPARs are key metabolic regulators, whose agonists are therapeutic targets for NAFLD/NASH currently under evaluation in phase I to III clinical trials, including the drug Pioglitazone (Boeckmans *et al*, 2019; Wu *et al*, 2020; Della Pepa *et al*, 2021). The time‐dependent decrease in PPAR emphasizes the importance to take proteome drift into account when evaluating PPAR agonist efficacy in hepatocyte models. This also applies for glycolysis/gluconeogenesis, lipid oxidation, and drug metabolic processes, which all decrease upon cell culture (Fig 6D).

Interestingly, hHSCs underwent gradual loss of vitamin A‐containing lipid droplets during primary cell culture—a typical feature of HSC activation. We confirmed this with our proteomics data, illustrated by an upregulation of the HSC activation marker α*‐*SMA by 30% at day‐7 compared to day‐1 (Fig 6F). HSC activation is a key event in fibrogenesis, during which quiescent HSCs differentiate into myofibroblast‐like cells and secret excessive extracellular matrix. This process is crucial for understanding the pathogenesis and development of liver fibrosis. Functional analysis of the more than 800 significantly changing proteins furthermore revealed increased expression in collagen microfibril organization, extracellular matrix organization and angiogenesis (Fig 6E). Specifically collagen type I, II, III, ECM1, as well as extracellular matrix modifiers Lysyl oxidase (LOXL2, LOXL4) had an about two‐fold increase at day 7 compared with day 1. Among these, LOXL2 has emerged as an attractive therapeutic target for inhibiting liver fibrosis (Barry‐Hamilton *et al*, 2010; Ikenaga *et al*, 2017). The collagenase MMP2, MMP14, and their inhibitors TIMP2 which also have anti‐fibrotic therapeutic potential were as well upregulated by 65–120%, likely reflecting a higher turnover rate of extracellular matrix (Craig *et al*, 2015; Chuang *et al*, 2019).

Several receptor tyrosine kinases and related proteins, known to be implicated in hepatic fibrosis or liver regeneration—including epidermal growth factor receptor (EGFR), fibroblast growth factor 2 (FGF2), and hepatocyte growth factor activator (HGFAC)—were also upregulated (Fig 6F). Liver disease such as cirrhosis can result in changes in the growth hormone‐insulin‐like growth factor axis (Donaghy *et al*, 2002; Bonefeld & Møller, 2011). In line with this, we observed about 50% higher levels of insulin growth factor binding proteins (IGFBP3, IGFBP4, and IGFBP7) by day‐7 upon HSC activation. Transforming growth factor (TGF)‐β is a major profibrogenic cytokine and targeting TGF‐β signaling has been explored to inhibit liver disease (Breitkopf *et al*, 2005; Rao & Mishra, 2019). Accordingly, TGF‐β receptors TGFBR1 and TGFBR2 levels were also more than 50% higher on day‐7 during HSC activation.

In previous studies, we had found that the peptidases ANPEP and DPP‐4, a well‐known drug target for T2D, were associated with NAFLD in a human cohort and mouse models (Niu *et al*, 2019), and we found them to be significantly and substantially upregulated upon HSC activation (Fig 6F). Furthermore, we had employed unbiased machine learning algorithms to select a panel of 14 circulating markers for predicting fibrosis in alcohol‐related liver disease (preprint: Niu *et al*, 2020). Levels of four of these proteins—all among the top predictors of fibrosis in ALD—increased 1.5 to 3‐fold, namely VCAM1, IGFBP7, IGFALS, and LGALS3BP. Thus, our dynamic proteomic profile of HSC activation provides functional insights into liver fibrosis and can allocate cellular origin of circulating markers.

Interestingly, “immune system process” and “leukocyte chemotaxis” were among the most highly enriched GO terms in upregulated proteins during cell culture of primary hHSCs (Fig 6E). The depth of our experiments allowed the identification of IL‐6, a protein of extremely low abundance in the plasma and generally considered to be secreted by macrophages such as the liver resident Kupffer cells and modulate hepatic inflammation (Schmidt‐Arras & Rose‐John, 2016; Han *et al*, 2020). Its levels doubled already by day‐3 of HSC culture. Altogether, more than 70 immune response proteins were upregulated, including the complement components (C3, C4A, C5, C8B) and proteins involved in regulation of leukocyte chemotaxis (VCAM1; Fig 6F). Thus, apart from ECM remodeling, HSC activation may perpetuate hepatic inflammation by secreting pro‐inflammatory factors to recruit leukocytes to the liver. These findings reveal a potential unappreciated role of HSC activation in the development of fibrogenesis.

In total, 342 proteins significantly decreased during primary culture of HSC (Dataset EV4). Among the significantly enriched KEGG pathways are “fatty acid biosynthesis” and “PPAR signaling pathway”. Decreased intensity in proteins related to fatty acid biosynthesis may reflect the loss of lipid droplet, one of the two prominent features of HSC activation, which we also observed morphologically under the microscope. As mentioned above, PPAR agonists have been investigated as possible therapeutic agents for liver disease. The expression of PPARγ is high in quiescent HSCs; however, PPARγ is suppressed during fibrosis process (Wu *et al*, 2020). Therefore, our observation of decreased protein levels in PPAR signaling confirms what is known but may also provide novel therapeutic targets, such as the proteins involved in lipid synthesis and degradation (FADS2, HMGCS1, FABP5, SCD, ACSL3).

### Dramatic proteome landscape shift in cirrhotic liver

**Figure 7:**
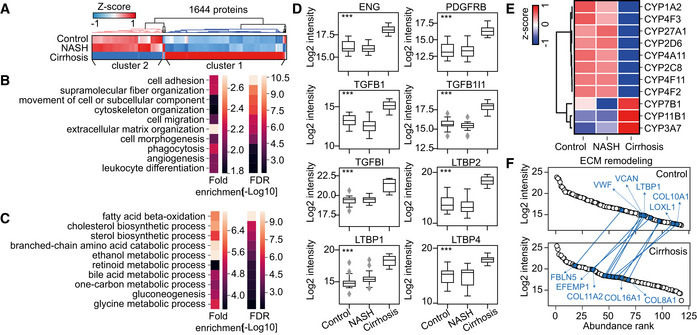
Liver proteome remodeling under pathological conditions Hierarchical clustering of proteins significantly differentially abundant between NASH (*n* = 20), cirrhosis (*n* = 10) and control groups (*n* = 15). Significance was calculated by ANCOVA, followed by Benjamini–Hochberg correction for multiple hypothesis testing (FDR < 0.05). Two major clusters of proteins were identified with Cluster 1 mainly upregulated in cirrhosis compared to NASH and controls and Cluster 2 downregulated. No additional replications of the experiment was done in laboratory.Ten representative highly enriched GOBP terms in proteins in Cluster 1 of Panel (A). Significance was calculated by Fisher’s exact test corrected for multiple hypothesis testing with FDR < 0.05.Ten representative highly enriched GOBP terms in proteins in Cluster 2 of Panel (A). Significance was calculated by Fisher’s exact test corrected for multiple hypothesis testing with FDR < 0.05.Box‐and‐whisker plot showing the distribution of log2‐intensity values of statistical significantly regulated proteins across three groups. Number of independent biological replicates is *n* = 15, 20 and 10 for the control, NASH and cirrhosis group, respectively. The black line in the middle of the box is the median, the top and the bottom of the box represent the upper and lower quartile values of the data and the whiskers represent the upper and lower limits for considering outliers (Q3+1.5*IQR, Q1−1.5*IQR) where IQR is the interquartile range (Q3–Q1). Significance was defined by ANCOVA followed by Benjamini–Hochberg correction for multiple hypothesis testing (FDR < 0.05) with a significance level of ***P < 0.001.Heatmap of CYP 450 family members that are statistically significantly abundant between three groups. Data were presented as mean protein abundance followed by *Z*‐score normalization across the three experimental groups. Number of replicates is same as Panel (C).Extracellular matrix (ECM) remodeling in liver cirrhosis. Upper and lower panel shows the abundance rank of proteins involved in ECM organization in the control (upper) and cirrhosis (lower) groups, highlighting top shifted ECM components in the cirrhosis group as compared with the control group.

Having generated an in‐depth human liver proteome, we used it to build a “spectral library”, to facilitate single‐run DIA analysis of liver biopsies in clinical cohorts. We applied this rapid and sensitive pipeline to investigate and compare various pathological conditions of the liver. Investigated liver samples were from cirrhosis patients requiring liver transplantation as the maximal end‐stage of CLD (*N* = 10), patients with morbid obesity and non‐alcoholic steatohepatitis requiring bariatric surgery (NASH, *N* = 20), and 15 healthy controls, of which 10 were obese but liver‐healthy. Participant characteristics can be found in Table EV6. In total, we quantified 6,475 protein groups (Dataset EV5). After filtering for at least 70% valid values in experimental groups, we obtained a data‐matrix with an average 5,382 proteins per sample and an overall data completeness of 91.8% (Methods). ANCOVA corrected for age and sex resulted in 1,644 proteins differentially abundant between cirrhosis, NASH, and healthy controls (Fig 7A, Dataset EV5). Of these, two thirds had increased abundance in liver cirrhosis and they are involved in extracellular matrix remodeling, signal transduction, cell morphogenesis and migration, immune response and angiogenesis (Fig 7B, Dataset EV5). These results provide the molecular basis for the clinical observations of liver cirrhosis, characterized by the replacement of normal liver tissue by scar tissue, the formation of new vessels leading to abnormal angioarchitecture in the cirrhotic liver, and the compromised immune system and dysregulated immune cell activation (Trebicka *et al*, 2019; Schierwagen *et al*, 2020). In particular, we found seven proteins that were significantly upregulated in cirrhotic liver biopsies to be uniquely detected in hepatic artery or portal vein in our in‐depth proteome atlas analysis just above (Fig 2A). Of these, tropomyosin alpha‐1 chain (TPM1) and Kinesin light chain 1 (KLC1) were detected in both but not in bulk liver. Both TGF‐β signaling and platelet‐derived growth factor signaling play an important role in mediating hepatic stellate cell activation and development of fibrosis. We found them to be upregulated in cirrhosis represented by the upregulation of PGDFRB, TGFB1 (TGF‐β1), TGFB1I1 (TGF‐β1 induced transcript 1 protein), TGFBI (TGF‐β induced protein ig‐h3), LTBP1, LTBP2, LTBP4, and ENG (transmembrane accessory receptor for TGF‐beta signaling; Fig 7D).

In contrast, down‐regulated proteins in liver cirrhosis are related to fatty acid metabolism, ethanol/drug metabolism, and retinol/retinoid metabolic processes reflecting metabolic impairment in patients with liver cirrhosis (Fig 7C). Decreased level of retinol/retinoid metabolic process likely reflect the loss of lipid‐storing phenotype, in particular the loss of retinyl ester‐containing lipid droplets in HSC—a key feature of HSC activation. Interestingly, while a majority of the CYP450 family enzymes are downregulated as expected, there are three exceptions—CYP3A7, CYP7B1 and CYP11B1 (Fig 7E). CYP3A7 is found predominantly in human fetal livers and is the major hepatic enzyme of the CYP450 family enzymes in the fetus (Li & Lampe, 2019). We detect an 18‐fold increase of CYP3A7 in cirrhotic liver compared to controls. This unexpected finding was consistent with a transcriptomics study in which expression of CYP3A7 was found to be slightly higher in HBV cirrhosis compared to normal livers while other CYP3A family members generally decrease (Chen *et al*, 2014). CYP7B1 is a crucial enzyme in the alternative bile acid synthetic pathway (Jia *et al*, 2021), and it has not been related to fibrosis. Similarly, little is known about the expression of CYP11B1 in liver cirrhosis, and we detect it to be upregulated by 77%. Thus, this study adds to our understanding of the CYP450 family members in liver diseases and provide new research directions for the pathogenesis and progression of liver diseases.

Hepatic fibrosis is characterized by excess accumulation and dynamic remodeling of ECM. Proteomics has the ability to comprehensively characterize the ECM molecular composition and its quantitative changes in liver fibrosis, which is essential for gaining insights into the mechanisms of liver disease. The altered ECM landscape in liver cirrhosis include the upregulation of collagens (type I, III, IV, V, VI, VIII, X, XI, XII, XIV, XV, XVI, XVIII, XXI), proteoglycans such as versican, decorin, lumican, and glycoproteins including fibulins, fibronectin, and laminins (Dataset EV5). Type X and XI collagens (COL10A1 and COL11A2) are the highest up‐regulated collagens, even though type I collagen (COL1A1, COL1A2) is the most abundant protein in the ECM, suggesting that not only the overall abundance but also the quantitative composition of the ECM constituents is altered in cirrhotic liver (Praktiknjo *et al*, 2018; Ortiz *et al*, 2021). To investigate this in a quantitative manner, we extracted all significantly elevated ECM associated proteins in cirrhotic liver and plotted their abundance rank in cirrhotic and healthy liver respectively which revealed 20 proteins whose abundance rank shifted by at least 20 (Fig 7F). COL10A1 had the most dramatic shift—from 114 in healthy control to 53 in cirrhotic liver, followed by EFEMP1, LOXL1, COL11A2, and FBLN5. In healthy liver, the ECM is constantly undergoing remodeling processes, by which components are being modified and degraded, tightly controlled to ensure homeostasis. Among the up‐regulated proteins associated with ECM organization were the matrix metalloproteinases MMP2, MMP14, MMP23A, ADAMTS5, and their tissue inhibitors (TIMP1, 3), as well as lysyl oxidase (LOXL1) which catalyzes collagen cross‐link formation, indicating increased collagen crosslinking. Interestingly, many of the overexpressed proteins such as collagens, Lysyl Oxidase, tissue metalloproteinase inhibitors were also upregulated upon hepatic stellate cell activation, indicating their potential cellular source.

Unlike the dramatic proteome shift in cirrhotic liver, NASH featured only marginal changes in liver proteome compared to normal livers characterized by 152 proteins significantly differentially abundant (Tukey *post hoc* test on ANCOVA significant hits with FDR < 0.05; Dataset EV5). NASH resulted in less proteome changes in the liver compared to cirrhosis, consistent with a transcriptomics study, in which only dozens of significantly differentially expressed proteins (DEPs) were detected in NASH and more than 1,000 DEPs in cirrhosis (Govaere *et al*, 2020).

**Figure 8:**
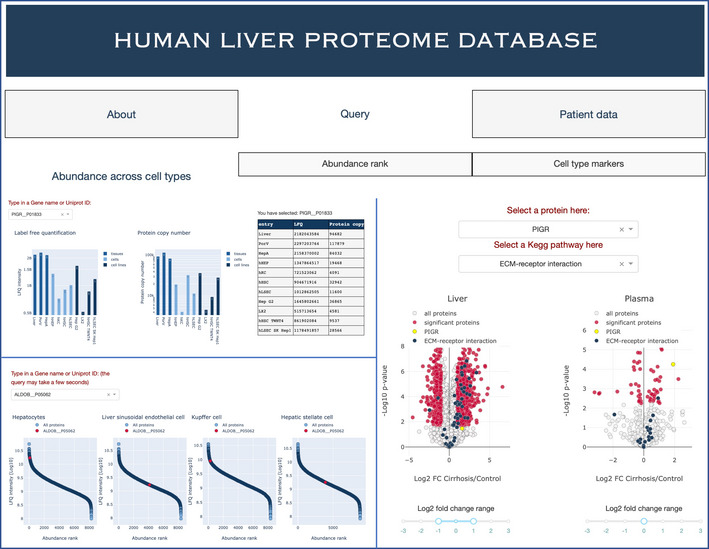
A web‐based dashboard app for data exploration The database enables inference of protein abundance across primary liver cell types and pathological conditions of liver disease.

To provide an open interface for easily accessing the data resource generated in this study, we built a web‐based Dashboard app that enables interactive data exploration and exportation (Methods, Fig 8). The database provides intuitive ways of data inference, such as: (i) proteome‐wide inference of protein abundance across liver tissue and cell types with both MS intensity and protein copy number, (ii) inference of protein abundance rank in a cellular proteome across four primary liver cell types, and (iii) inference of proteome changes in liver and plasma upon pathological conditions such as liver cirrhosis at protein and pathway level. In addition, the database provides information of observed peptides for each identified protein, which can be useful for building targeted assay such as parallel reaction monitoring. The database can be accessed at www.liverproteome.org.

## Discussion

The liver is a vital organ responsible for hundreds of functions in the body. Multiple risk factors predispose to liver diseases, which imposes a huge burden on the global health systems (Loomba & Sanyal, 2013). A better understanding of basic liver biology and the underlying mechanism of pathology can aid prevention, diagnosis, and treatment. In the era of “big data” when omics data are increasingly being generated in human cohorts, a fast and sensitive proteomics workflow for liver tissue is required.

MS‐based proteomics is a constantly developing field. Through strategies such as multi‐enzyme digestion, extensive fractionation, the use of various fragmentation techniques, and matching to other tissue proteomes, current technology has been able to identify > 10,000 proteins in bulk human liver tissue at the expense of analysis time (Bekker‐Jensen *et al*, 2017; Wang *et al*, 2019). However, these studies did not provide quantitative information at the cell type level. In comparison to bulk liver tissue, there are relatively few primary liver cells in isolates from an individual, requiring more efficient sample preparation procedures and more sensitive mass spectrometry acquisition methods. To address these challenges, we adopted state‐of‐the‐art technologies—including the loss‐less high‐pH reversed‐phase fractionation (Kulak *et al*, 2017). This allowed us to quantify the largest cell type‐resolved quantitative human liver proteome atlas, consisting of 10,528 proteins assembled from 158,670 sequence‐distinct peptides. The largest prior efforts investigated primary cells and only at one time point, identifying 6,788 proteins (Sun *et al*, 2010) and 9,791 proteins (Ölander *et al*, 2020).

Data‐independent acquisition (DIA) in MS‐based proteomics has demonstrated superior performance in recent years in terms of proteome depth, data completeness, and quantification reproducibility as compared to data‐dependent acquisition (DDA), and has become the preferred method in clinical studies (Bruderer *et al*, 2015; Guo *et al*, 2015; Karayel *et al*, 2020; Hansen *et al*, 2021). To achieve an optimal data quality, a deep and high‐quality peptide spectral library is typically generated for a specific type of tissue. However, this step is laborious and not every group is equipped with the necessary instrumentation and technology to generate high‐quality spectral libraries. The extensive dataset generated in our study can serve as a spectral library for high‐throughput proteomic analysis of patient samples. With an optimized DIA method integrated with the advanced signal‐processing algorithm ΦSDM (Grinfeld *et al*, 2017), and MaxQuant.Live for direct instrument control (Wichmann *et al*, 2019), we achieved proteome coverage of 6,000 proteins in 100 min measurement time in single‐runs, demonstrating suitability of this workflow for analyzing clinical samples.

We investigated multiple dimensions of the human liver proteome: (i) four isolated primary liver cell types and three tissue types, (ii) dynamic proteome profiles during primary cell culture, and (iii) changes in the liver proteome of a cohort of patients with NASH and end‐stage liver cirrhosis. The resulting quantitative data and its in‐depth bioinformatics analysis highlighted new quantitative aspects of basic cell and tissue biology. For example, we estimate—using the “proteomic ruler” approach (Wisniewski *et al*, 2014)—that half of the protein mass in hepatocytes is composed of enzymes and about 1.2 of 4 billion protein molecules in each cell carry out metabolic pathway functions, reflecting their extremely high metabolic activity. Furthermore, there are 15–77 million protein copies in OXPHOS complexes I–IV in hHEPs, up to six‐fold higher than other cell types. In contrast, less than 20% of protein mass in hLSECs is dedicated to enzymatic function, but significantly more to transporters, ribosomal proteins, cytoskeleton, and extracellular matrix proteins, reflecting higher transcytosis and the functional demands for regulating sinusoidal blood flow (Poisson *et al*, 2017).

These cell type level quantitative information can also help elucidate disease mechanisms and provide useful information for drug development. Our copy number catalogue contains quantitative information of disease‐relevant proteins, such as those known to carry SNPs predisposing or protecting against NAFLD, including PNPLA3, GCKR, and HSD17B13 (all most abundant in hHEPs). MBOAT7 is a transmembrane protein involved in phosphatidylinositol remodeling, and its rs641738C>T variant, strongly contributes to pathogenesis of a range of liver diseases in human genetic studies (Buch *et al*, 2015; Thabet *et al*, 2016, 2017). We find its copy numbers to be 25‐fold higher in hHSCs compared to hHEPs. In view of the key role of hHSC in fibrogenesis, this raises interesting mechanistic questions. Among therapeutically relevant proteins, TGF‐beta receptor and PDGF receptor have highest levels in hHSCs but are also present in all other liver cell types examined. Knowledge of cell type specific abundance should be useful for cell type‐specific targeting, which could be more effective than global approaches with less adverse effects (Klein *et al*, 2012, 2019). Furthermore, our set of more than 100 proteins uniquely detected in one of the cell types, may serve as protein targets in developing targeted drug delivery methods or as new markers for isolating liver cell types. We found that cross‐referencing the bulk and single‐cell RNA sequencing data as well as the image collection generated by the Human Protein Atlas project helps to validate cell type specificity of the markers in a scalable manner.

Human primary cell culture are common systems for drug evaluation and development (Eglen & Reisine, 2011), but researchers need to know how close they mimic the *in vivo* situation, especially after culturing. In this regard, we previously observed significant changes in the proteome of murine primary hepatocytes upon cell culture (Azimifar *et al*, 2014). Consistent with this, we here observed extensive proteome remodeling upon cell culture with 41% hepatocyte proteins changing significantly in 7 days. It is characterized by reduced levels of metabolic or energy homeostatic processes, including PPAR signaling—a pathway regulating lipid metabolism and inflammation, and an anti‐NASH drug target under active development (Gross *et al*, 2017; Boeckmans *et al*, 2019; Wu *et al*, 2020). These changes should be considered when evaluating drug efficacy in cell models. Investigating dynamic proteome changes upon HSC activation can help in the development of therapies against liver fibrosis (Zhang *et al*, 2017). Our time course experiment revealed the dynamics of this process, including the upregulation of proteins involved in ECM organization and immune response. Our finding of upregulated immune response may indicate a new role of HSC in promoting inflammation and immune responses in liver disease.

To connect our findings from human tissue and dynamic primary cell culture directly to patients, we investigated the liver proteome changes in NASH and cirrhosis in a cohort of 45 individuals. Previously, we have observed dramatic remodeling in the plasma proteome of patients with liver cirrhosis, whereas only a few proteins significantly changed in NAFLD (Niu *et al*, 2019). In line with this, only a handful proteins in the liver significantly changed in NASH, whereas one third of the total liver proteome was significantly altered in liver cirrhosis, including increased levels of signaling pathways, extracellular matrix components and immunological response, as well as decreased levels in various metabolic pathways. This agrees with the fact that NASH is an intermediate but progressive form of chronic liver disease (Simon *et al*, 2021), whereas liver cirrhosis is the common end stage of a wide variety of chronic liver diseases, characterized by substantial structural changes and impaired liver functions. This important insight agrees with the reversibility of NASH and underlines that therapeutic approaches for this stage may be more successful than in cirrhosis, in which the proteomic and thereby structural changes are very pronounced. Large portions of NAFLD and NASH do not present with liver‐related events and this paper strengthens the rationale for approaches to improve the large regenerative capacity still available in NASH patients. By contrast, cirrhosis has a very high liver‐related mortality, rendering regeneration of the liver very improbable in the late stages, when complications of cirrhosis such as refractory ascites and acute‐on‐chronic liver failure occur. Our data and analyses are also valuable for cirrhosis research. For instance, the lack of important secretory capacity may point to therapeutic opportunities aiming at replacement of vital substances such as haptoglobin. This has only been done for albumin (Fernández *et al*, 2020) and coagulation factors (Bedreli *et al*, 2016) but not for haptoglobin. Our detailed catalogue of significantly altered proteins and pathways in the disease cohort and the primary cell course experiment provide a valuable tool for the research community to interpret data and discover new therapeutic targets. As an example, many of the proteins upregulated in bulk cirrhotic liver tissue can be mapped to hepatic stellate cell activation, highlighting intervention points before full blown cirrhosis. This can be used in conjunction with circulating biomarkers identified by proteomics such as the ones we previously showed for NAFLD and ALD in plasma (Niu *et al*, 2019; preprint: Niu *et al*, 2020).

A limitation to this study is that we cannot exclude the possibility of some blood contamination in the sampling of liver biopsies, hepatic artery, and portal vein even though we rinsed and flushed the hepatic vessels upon sampling. Liver perfusion would be a good way to remove blood contamination; however, it is only practical in mouse models.

To facilitate interactive data visualization of the large proteomics data sets, we built a dashboard application using the open source Dash framework in Python. We have also integrated pathological proteome changes in liver and plasma, which we generated in previous studies, allowing users to explore proteome changes at the level of both proteins and KEGG pathways. We hope that these data will become a valuable resource for basic, translational and clinical research focusing on liver pathophysiology, biomarker discovery and drug development.

## Materials and Methods

### Reagents and Tools table

**Table:** 

Reagent/Resource	Reference or Source	Identifier or Catalog Number
**Experimental Models**		
Isolated primary human liver cell types	Samsara sciences (San Diego, CA, USA)	Donors: HL170051, HL170063, HL160034
Human hepatic artery, portal vein, liver tissues	(Rheinwalt *et al*, 2020); (Schierwagen *et al*, 2020)	N/A
Human liver cell lines	Trebicka Lab	N/A
**Chemicals, enzymes, and other reagents**		
Water, Optima™ LC/MS Grade	Fisher Chemical	Cat # W64
Water, HiPerSolv CHROMANORM^®^ for LC‐MS	VWR Chemicals (supplier)	Cat # 83645.290
25% LC‐MS grade ammonia	Merck Millipore	Cat # 533003
sodium deoxycholate (SDC) reduction and alkylation buffer	PreOmics GmbH	Cat # P.O.00032 iST
Tryptophan	Sigma Aldrich	Cat # T8941
Trypsin	Sigma Aldrich	Cat # T6567
Isopropanol	Sigma Aldrich/Merck	Cat # 67‐63‐0
Formic acid	Sigma Aldrich/Merck	Cat # 64‐18‐6
Acetonitrile	Sigma Aldrich/Merck	Cat # 75‐05‐8
Trifluoroacetic acid	Sigma Aldrich/Merck	Cat # 76‐05‐1
LysC	Wako	Cat # 129‐02541
DMEM cell culture medium	Gibco	Cat #11995073
Medium 199	Lonza	Cat # BE12‐117F
RPMI cell culture medium	Gibco	Cat # 21875‐034
William's Medium E	Life Technologies	Cat #A12176‐01
febal bovine serum (FBS)	Gemini Bio	Cat #100‐106
Penicillin/Streptomycin	Life Technologies	Cat #15240‐062
trypsin/EDTA	Sigma Aldrich/Merck	Cat # T3924
Phosphate Buffered Saline (PBS)	Gibco	Cat # 14190‐094
Insulin‐Transferrin‐Selenium (ITS)	Life Technologies	Cat # 41400‐045
10^−7 ^M Dexamethasone	Lonza	Cat #CC‐4150DD
EGM‐2 endothelial cell growth basal medium	Lonza	Cat # CC‐4176
HEPES	Biochrom	Cat #L1613
EBM‐2 endothelial cell growth basal medium	Lonza	Cat # CC‐3156
**Software**		
MaxQuant (1.5.3.30)	https://maxquant.org/	N/A
MaxQuant.Live (1.0)	MaxQuant.Live	N/A
Spectronaut (13.3 and 14.1)	https://biognosys.com/software/spectronaut/	N/A
Perseus (1.5.5.5 and 1.6.5.0 )	https://maxquant.net/perseus/	N/A
Python (3.7)	python.org	N/A
Jupyter Notebook	https://jupyter.org/	N/A
Cytoscape (2.3.1)	https://cytoscape.org/	N/A
**Other**		
Bravo Automated Liquid Handling Platform	Agilent	Cat # G5409A
Bioruptor Plus sonication device	Diagenode	Cat # B01020001
Reprosil‐Pur Basic C18, 1.9 µm	Dr. Maisch Gmbh	Cat # r119.b9
ThermoMixer^®^	Eppendorf	Cat # 460‐0223
Concentrator plus	Eppendorf	Cat # F‐45‐48‐11
Silicone sealing mat, for 96 well PCR‐plates	Nerbe Plus	Cat # 04‐090‐0000
PicoFrit self‐pack columns	Pico FRIT	Cat # PF360‐75‐15‐N‐5
iST' sample preparation kit	PreOmics GmbH	Cat # P.O. 00001
high‐pH reversed phase fractionator (SPIDER)	PreOmics GmbH	N/A
Empore SPE SDB‐RPS disk	Sigma Aldrich/Merck	Cat # 66886‐U
PRSO‐V2 column oven	Sonation	N/A
96‐Well Plates	Thermo Fisher	Cat # AB‐1300
NanoDrop™ One/OneC Microvolume UV‐Vis Spectrophotometer	Thermo Fisher	Cat # ND‐ONEC‐W
EASY‐nLC™ 1200 System	Thermo Fisher	Cat # LC140
Orbitrap Exploris 480 Mass Spectrometer	Thermo Fisher	Cat # BRE725533
FAIMS Pro™ Interface	Thermo Fisher	Cat # FMS02‐10001
Costar^®^ 6‐well	Corning	Cat # 3516
Q Exactive™ HF‐X Hybrid Quadrupole‐Orbitrap™ Mass Spectrometer	Thermo Fisher	Cat # 0726042

### Methods and Protocols

#### Experimental model and subject details

##### Cell lines

Snap‐frozen cells were thawed in a water bath at 37°C and transferred to culture medium. Viability was controlled and was systematically over 95%. For cryopreservation, cells were centrifuged at 200 *g* for 5 min. LX2 cells and TWNT‐4 cells were grown with cell culture medium (DMEM + 20% FCS + Penicillin/Streptomycin) in 250 ml plastic flasks in a humidified 5% CO_2_ incubator at 37°C. After reaching 80% confluency, cells were passaged with a 1:3 split ratio. Detachment was achieved by incubating the cells with 0.05% trypsin/EDTA solution (solved in phosphate buffered saline (PBS)) for 5 min at 37°C. HepG2 cells were grown with cell culture medium (DMEM + 10% FCS + Penicillin/Streptomycin) in 250 ml plastic flasks in a humidified 5% CO_2_ incubator at 37°C. Cells were seeded at density of about 3 × 10^6^ cells/80 cm^2^. After reaching 80% confluency, cells were passaged with a 1:2 split ratio. SK Hep1 cells were cultured in a humidified 5% CO_2_ incubator at 37°C. Cells were grown in culture medium (M199 + 10% FCS + Penicillin/Streptomycin). Cells were plated at density of about 4 × 10^6^ cells/80 cm^2^ in flasks coated with collagen IV. After reaching 80% confluency, cells were passaged with a 1:10 split ratio. Detachment was achieved by incubating the cells with 0.05% Trypsin/EDTA solution (solved in PBS) for 5 min at 37°C.

##### Primary cells

Primary human HSC, KC, HEP, and LSEC were obtained from Samsara sciences (San Diego, CA, USA). The cells used in the comparative analysis of basal proteome between the four cell types have not been previously cultured after isolation. Characteristics and purity of the isolated cells were assessed by the provider (liver cell characterization attached as Appendix). For the time course primary cell culture experiment, snap‐frozen primary cells were thawed in a water bath at 37°C and transferred to culture medium. Viability was controlled and was systematically over 95%. For cryopreservation, cells were centrifuged at 200 *g* for 5 min. Primary human HSC were grown in cell culture medium (DMEM + 10% fetal bovine serum (FCS) + Penicillin/Streptomycin) in a humidified 5% CO_2_ incubator at 37°C. Cells were seeded at density of about 6,370 cells/cm^2^. After reaching 85% confluency, cells were passaged with a 1:3 split ratio. Detachment was achieved by incubating the cells with 0.05% Trypsin/EDTA solution (solved in PBS) for 5 min at 37°C. Primary human KC were grown in cell culture medium (RPMI 1640 + 10% FCS + Penicillin/Streptomycin) in a humidified 5% CO_2_ incubator at 37°C. Cells were plated at a concentration of 0.3 × 10^6^ cells/ml. Primary human HEP were grown in cell culture medium (Williams' Medium E + 1% Insulin‐Transferrin‐Selenium (ITS) + 10^−7^ M Dexamethasone + Penicillin/Streptomycin + 10 mM HEPES) in a humidified 5% CO_2_ incubator at 37°C. Primary human LSEC were grown in cell culture medium (EBM‐2 + EGM‐2 endothelial cell growth basal medium singlequots) in a humidified 5% CO_2_ incubator at 37°C. Cells were plated at a seeding density of about 3,000 cells/cm^2^. Cells were plated in flasks coated with collagen I.

##### Human tissues

Liver tissue, hepatic artery, and portal vein which were used to generate the fractionated, deep proteomes were collected from three receivers and three donors of liver transplantation during 2001 and 2003. For the human cohort (*N* = 45 in total), additional 10 liver samples from patients with cirrhosis requiring liver transplantation (performed between 2001 and 2003) were used. Patients with liver cirrhosis were classified as Child‐Pugh class B (*n* = 4) or C (*n* = 6) with a median score of 10 and had a median MELD (model of end‐stage liver disease) score of 18.5 (minimum 13, maximum 23). Liver samples of patients with morbid obesity and non‐alcoholic steatohepatitis (NASH, *n* = 20), as well as samples from obese but liver‐healthy individuals (*n* = 10) were collected during bariatric surgery, which was performed at the Department of Bariatric, Metabolic and Plastic Surgery, St. Franziskus‐Hospital Cologne, Germany, between July 2018 and May 2019 (Rheinwalt *et al*, 2020). Additional five liver samples from healthy donors of liver transplantation (performed between 2001 and 2003) were used as healthy controls, adding to 15 in total. Patients with NASH had a median fibrosis stage of F2 (minimum F1, maximum F3; Kleiner *et al*, 2005), a median NAFLD activity score (NAS) of 6 (minimum 5, maximum 7 on a scale of 0–8) and had at least perisinusoidal fibrosis. Liver fibrosis stage from biopsy: F1/2/3 = 4/14/2. NAFLD activity score: 5/6/7 = 3/15/2. Liver steatosis score: 1/2/3 = 1/14/5. Hepatocyte ballooning score: 1/2 = 1/19. Lobular inflammation score: 1/2 = 4/16. Obese but liver‐healthy control individuals had less than 5% of parenchymal steatosis. The diagnosis was performed independently by two experienced pathologists as described elsewhere (Schierwagen *et al*, 2020). Samples were washed with ice‐cold PBS, snap‐frozen in liquid nitrogen and stored in −80°C after collection. The investigation was approved by the ethical committee of the University of Bonn (document no. 029/13 and 194/17, respectively) by the ethics committees of the regional Medical Association Nordrhein (project identification code 2017110) in accordance with the Declaration of Helsinki. All patients signed an informed consent before being enrolled in the study.

##### Sample preparation for MS analysis

Tissue samples were ground to a frozen powder using a mortar and pestle in liquid nitrogen. Powdered samples were then resuspended in 350 μl of sodium deoxycholate (SDC) reduction and alkylation buffer (PreOmics GmbH, Martinsried, Germany) and boiled for 10 min while vortexing at 1,200 rpm in the thermomixer to denature proteins (Kulak *et al*, 2014). The lysates were sonicated at full power for 30 cycles with 30 s intervals using a water bath sonicator (Diagenode Bioruptor^®^, Liège, Belgium). Protein content was determined by Tryptophan assay. An aliquot of 150 μl homogenate was digested overnight with LysC and trypsin in a 1:50 ratio (μg of enzyme to μg of protein) at 37°C and 1,700 rpm in the thermomixer. On the following day, boiling and sonicating was repeated followed by an additional step of digestion for 2 h (1:100 ratio). Peptides were acidified to a final concentration of 0.1% trifluoroacetic acid (TFA) to quench the digestion reaction. Peptide concentration was estimated using Nanodrop and 20 μg of peptides was loaded on two 14‐gauge Stage‐Tip plugs. Peptides were washed first with isopropanol/1% TFA (200 μl) and then 0.2% TFA (200 μl) using an in‐house‐made Stage‐Tip centrifuge at 2,000 *g*. Peptides were eluted with 60 μl of elution buffer (80% acetonitrile/1% ammonia) and dried at 60°C using a SpeedVac centrifuge (Eppendorf, Concentrator plus). Dried peptides were redissolved and sonicated in 5% acetonitrile/0.1% TFA, and concentration was measured using Nanodrop. About 50 μg of peptides were fractionated into eight fractions using basic reverse phase high‐pH fractionation with the SPIDER fractionator (PreOmics GmbH, Martinsried, Germany). Cell lines, isolated and cultured human primary cells were processed similarly to the tissue samples without liquid nitrogen crushing.

##### LC‐MS/MS

All samples were measured using LC‐MS instrumentation consisting of an EASY‐nLC 1200 system (Thermo Fisher Scientific, San Jose, CA) interfaced on‐line with a Q Exactive HF‐X Orbitrap or Orbitrap Exploris 480 equipped with a FAIMS Pro Interface (Thermo Fisher Scientific, Bremen, Germany). Samples were prepared and measured in a randomized manner to avoid systematic bias. No blinding was performed. The latter LC‐MS instrumentation setup was used in the time‐course experiment of primary cell culture. For all samples, purified peptides were separated on 42.5 cm HPLC‐columns (ID: 75 µm; in‐house packed into the tip with ReproSil‐Pur C18‐AQ 1.9 µm resin (Dr. Maisch GmbH)). For each LC‐MS/MS analysis, around 0.5 µg peptides were injected for the 100 min gradients. Peptides were loaded in buffer A (0.1% formic acid) and eluted with a linear 82 min gradient of 3–23% of buffer B (0.1% formic acid, 80% (v/v) acetonitrile), followed by a 8 min increase to 40% of buffer B. The gradients then increased to 98% of buffer B within 6 min, which was kept for 4 min. Flow rates were kept at 350 nl/min. Re‐equilibration was done for 4 μl of 0.1% buffer A at a pressure of 980 bar. Column temperature was kept at 60°C using an integrated column oven (PRSO‐V2, Sonation, Biberach, Germany).

MS spectra of fractionated samples were acquired with a Top15 data‐dependent MS/MS scan method (DDA, topN method). Target values for the full‐scan MS spectra was 3e+6 in the 300–1,650 m/z range with a maximum injection time (IT) of 25 ms and a resolution of 60,000 at m/z 200. Precursor ions targeted for fragmentation were isolated with an isolation width of 1.4 m/z, followed by higher‐energy collisional dissociation (HCD) with a normalized collision energy of 27 eV. Precursor dynamic exclusion was activated with 30 s duration before triggering the subsequent scan. MS/MS scans were performed at a resolution of 15,000 at m/z 200 with an automatic gain control (AGC) target value of 1e+5 and an IT of 25 ms.

MS spectra of unfractionated patient samples were acquired in single‐shot with a data‐independent acquisition (DIA) method, enabled by MaxQuant.Live (Wichmann *et al*, 2019) in which the scan protocol was defined. Each acquisition cycle was consisted of a survey scan at resolution of 60,000 with an AGC of 3e+6 and IT of 100 ms, followed by 66 DIA cycles at resolution of 15,000 with an AGC of 3e+6 and IT of 22 ms at range 300–1,650 m/z (Table EV7). HCD fragmentation was set to normalized collision energy of 27%. In all scans, PhiSDM (Grinfeld *et al*, 2017) was enabled with 100 iterations, spectra type was set to centroid.

For spectra acquisition in the time course experiment of primary cell culture, a FAIMS Pro Interface was mounted between the electrospray ionization source and the mass spectrometer (Orbitrap Exploris 480). The ion source was set to a voltage of 2,650 (V) in positive ion mode with an ion transfer tube temperature of 275°C. FAIMS mode was set to “standard resolution” with a total carrier gas flow of 4.6 l/min throughout the entire acquisition period. For single‐shot analysis, MS spectra were acquired using DIA mode with intra‐analysis compensation voltage (CV)‐switching. Each acquisition cycle was consisted of a survey scan at resolution of 120,000 with a normalized AGC target (%) of 300% and 28 ms of injection time at scan range of 350–1,650 m/z, followed by 22 DIA cycles at resolution of 15,000 with a normalized AGC target (%) of 3,000% and 25 ms of injection time repeated for three CVs (−40 V, −55 V, and −70 V), totaling a cycle time of around 3 s (Table EV8). HCD fragmentation was set to normalized collision energy of 30%. To create a spectral library based on gas‐phase fractionation, a pooled, unfractionated primary cell sample for each cell type was analyzed in single‐shots using seven methods of different CVs stepping from −40 V to −70 V with an increment of −5 V. These gas‐phase fractionation methods consisted of a survey scan at resolution of 120,000 followed by 66 DIA scans at resolution of 15,000 (Table EV8). The resulting MS spectra were analyzed together with data acquired by the above‐described single‐shot method of intra‐analysis CV switching to boost identifications.

##### MS data processing

All raw files of fractionated samples were analyzed by MaxQuant v.1.5.3.30 software (Cox & Mann, 2008) using the integrated Andromeda Search engine (Cox *et al*, 2011) and searched against the Uniprot human database (April 2017 release including isoforms and sequence variants). Enzyme specificity was set to trypsin with a maximum of two missed cleavages. The search included cysteine carbamidomethylation as fixed modification and oxidation on methionine and N‐terminal acetylation as variable modifications with a minimum length of seven amino acids. A false discovery rate (FDR) of 1% was set to PSM and protein levels. The “match between runs” algorithm was activated to transfer MS/MS identifications between runs where applicable (Nagaraj *et al*, 2012). Label‐free quantification was performed with the integrated MaxLFQ algorithm using a minimum ratio count of 2 (Cox *et al*, 2014). A spectral library was generated from the fractionated samples for single‐injection DIA analysis of patient samples in the clinical cohort (*N* = 45).

All raw files of patient samples were analyzed by Spectronaut (version 13.3) with default settings except that the normalization strategy for “cross‐run normalization” was wet to “local normalization” based on rows with “Qvalue complete” (Bruderer *et al*, 2015). A FDR of 1% was set to peptide precursor level and 1% to protein level. The FDR method of Storey was used (Storey & Tibshirani, 2003). The library generated from fractionated samples described above was used in the targeted analysis of single‐shot DIA data against the human Uniprot fasta database (January 2018 release including isoforms and sequence variants).

All raw files in the time course experiment of primary cell culture were analyzed by Spectronaut (version 14) in directDIA mode against the human Uniprot fasta database (January 2018 release including isoforms and sequence variants). Default settings were used except that the normalization strategy for “cross‐run normalization” was wet to “local normalization” based on rows with “Qvalue complete”.

For the PCA, no pre‐filtering was applied due to the diversity of sample types and relatively small number of biological replicates. Missing values were imputed by drawing from a down‐shifted normal distribution relative to that of a sample’s proteome abundance distribution (down‐shifted mean by 1.8 standard deviation (s.d.) and scaled s.d. (0.3)). The same imputation method was applied throughput all analyses. For the proteome comparison across four primary cell types (fractionated samples), we filtered proteins quantified in this study for at least two valid values in three biological replicates of at least one cell type, resulting in 8,866 proteins. For the patient samples, we filtered proteins quantified in the cohort for at least 70% valid values at experimental group level (healthy, NASH and cirrhosis group), resulting in 5,843 proteins with an average of 5,382 proteins per sample and an overall data completeness of 91.8%. For comparison between time points of the same cell type in the time course experiment of primary cell culture, we filtered proteins for at least two valid values in three biological replicates of at least one time point within each cell type, resulting in total number of proteins ranging between 6,100 and 6,900 proteins in the three cell types.

##### Bioinformatics and statistical analysis

Statistical and bioinformatics analysis was performed with the Perseus software (version 1.6.2.1) and Python software. No samples analyzed were excluded for the down‐stream statistical and bioinformatics analysis. One‐way ANOVA and ANCOVA significance level was controlled with FDR below 5% with Benjamini–Hochberg for multiple hypothesis testing. GO Enrichment Analysis in the primary cell type proteome was performed with ClueGo (Bindea *et al*, 2009), a plug‐in app in Cytoscape (Shannon *et al*, 2003), with default settings except the following changes: Ontologies file of Biological Process was downloaded from EBI‐Uniprot on September 04, 2018 which contains 15,947 terms with 17,940 available unique genes. Customed reference set which contains 9,223 unique genes (quantified in the uncultured primary liver cell types in this study) was used in Fisher’s exact test. Term significance was corrected by Benjamini–Hochberg with a FDR of below 1%. GO tree levels was controlled at 2–3 for HEP and KC, and 2–4 for LSEC and HSC with a threshold of 10 genes and 10% of genes per term to maximize the information presentable. Both GO term fusion and grouping are activated. ANCOVA controlled for age and sex was performed to determine differentially abundant proteins across the three groups of patient samples: NASH, healthy control, and patients with liver cirrhosis. Normality and equality of variance were assessed using the Pingouin statistical package in Python, resulting in 86% of the proteins having equal variance between the three experimental groups and 75% of proteins having a normal distribution (*P* < 0.05). GO Enrichment Analysis in patient samples between NASH, cirrhosis and control was performed with the online Gene Ontology Resource (geneontology.org). Protein class annotation was downloaded from the Human Protein Atlas (HPA) database (Uhlen *et al*, 2016). The interactive dash board application was built using the open source web application framework of Plotly Dash and Python.

## Author contributions

**Matthias Mann:** Conceptualization; Resources; Software; Supervision; Funding acquisition; Investigation; Visualization; Methodology; Writing—original draft; Project administration; Writing—review and editing. **Lili Niu:** Conceptualization; Data curation; Formal analysis; Investigation; Visualization; Methodology; Writing—original draft; Project administration. **Philipp E Geyer:** Conceptualization; Supervision; Methodology; Writing—review and editing. **Rajat Gupta:** Investigation; Writing—review and editing. **Alberto Santos:** Methodology; Writing—review and editing. **Florian Meier:** Methodology; Writing—review and editing. **Sophia Doll:** Investigation. **Nicolai J Wewer Albrechtsen:** Writing—review and editing. **Sabine Klein:** Resources; Data curation. **Cristina Ortiz:** Resources; Data curation. **Frank E Uschner:** Resources; Data curation. **Robert Schierwagen:** Resources; Data curation; Investigation; Writing—review and editing. **Jonel Trebicka:** Conceptualization; Resources; Funding acquisition; Project administration; Writing—review and editing.

In addition to the CRediT author contributions listed above, the contributions in detail are:

LN acquired and interpreted the proteomics data, designed and developed the project, and wrote the manuscript. PEG designed the project, performed proteomic sample preparation and edited the manuscript. RG prepared proteomics sample and edited the manuscript. AS guided the data analysis and revised the manuscript. FM optimized proteomic acquisition method. SD performed proteomic sample preparation. NJWA critically reviewed and edited the manuscript. SK, CO, FEU and RS performed cell culture and edited the manuscript. JT designed the project, provided all biological and clinical materials, and edited the manuscript. MM designed and supervised the project, interpreted the data and edited the manuscript.

## Disclosure and competing interests statement

The authors declare that they have no conflict of interest. Matthias Mann is an editorial advisory board/EMBO Member. This has no bearing on the editorial consideration of this article for publication.

## Supporting information



Appendix

Expanded View Figures PDF

Dataset EV1

Dataset EV2

Dataset EV3

Dataset EV4

Dataset EV5

Table EV1

Table EV2

Table EV3

Table EV4

Table EV5

Table EV6

Table EV7

Table EV8

## Data Availability

The datasets and computer code produced in this study are available in the following databases: The mass spectrometry proteomics data have been deposited to the ProteomeXchange Consortium via the PRIDE (Vizcaíno *et al*, 2014) partner repository with the dataset identifier PXD027722. The code generated in this study have been uploaded to the GitHub repository https://github.com/llniu/Human_Liver_Proteome. The interactive dash board application can be accessed at www.liverproteome.org.
