# Supplementary material for: Dynamic human liver proteome atlas reveals functional insights into disease pathways
Source: Mol Syst Biol. 2022 May 17;18(5):e10947. doi: 10.15252/msb.202210947 (PMC9112488; doi:10.15252/msb.202210947)

Table of Contents

Hepatic stellate cell characterization SAM-PI HL170051SC V2 2

Liver sinusoidal endothelial cell characterization SAM-PI HL170051EC V2 3

HL160034 Donor history report 5

HL170051 Donor history report 7

HL170063 Donor history report 9

PRODUCT INFORMATION SHEET

Lot: HL170051SC

FOR RESEARCH USE ONLY

Cell Type: Adult human hepatic stellate cells

Post thaw yield:

Passage 0-1: > 0.60 x 10^6 cells per vial

Passage 2-4: > XX x 10^6 cells per vial

Population doubling time: XX days

Post thaw viability: > 65%

Culture morphology: Shown for passage p1 cells

<50% Confluent

>85% Confluent

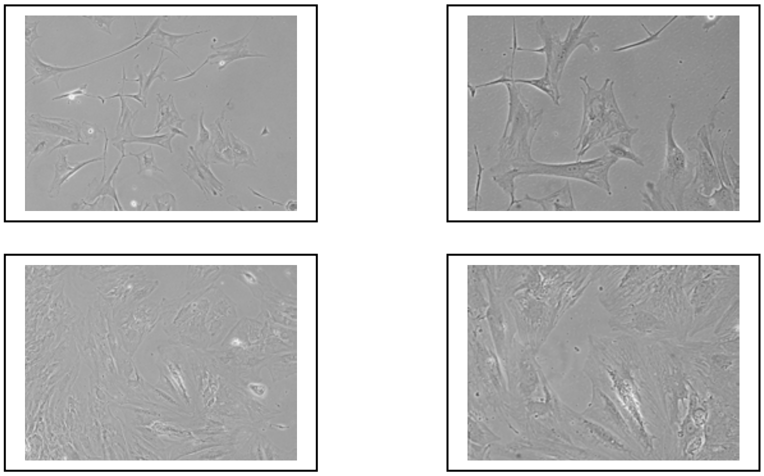

100X 200X

Phenotype in culture:

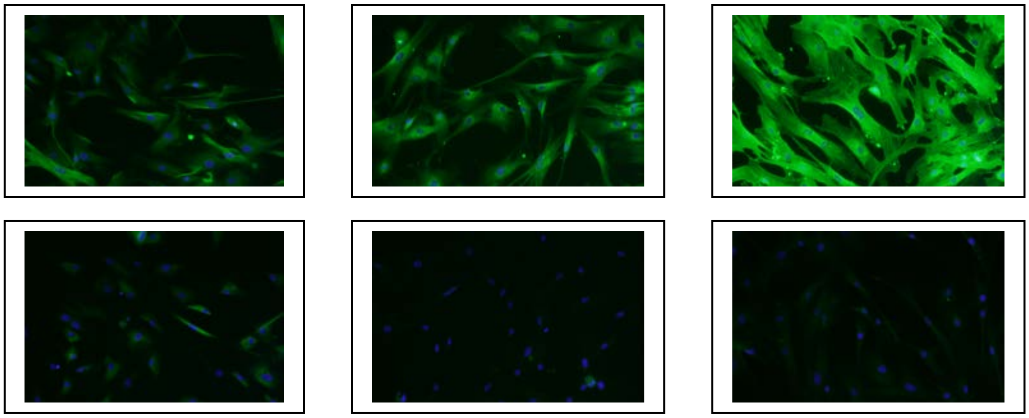

Fluorescent Staining Shown at passage p1 (200x). Desmin: Thermo #RB-9014-P0, 1:200; GFAP: Abcam ab7260, 1:200; SMA Abcam ab5694, 1:200; TE-7 Millipore CBL271, 1:300.

Results above determined by following Samsara stellate cell care and handling protocols

Samsara Sciences, Inc.
6310 Nancy Ridge Dr., Ste. 106
San Diego, CA 92121
Phone: 858-617-0790
www.samsarasciences.com
info@samsarasciences.com

PRODUCT INFORMATION SHEET
Human Liver Endothelial Cells
Lot: HL170051EC
For Research Use Only

Cell Type: Adult human liver endothelial cells
Post thaw yield: > 1.0 x 10^6/vial
Average cell size: 20.4 microns

Cells provided: Cryopreserved post isolation
Post thaw viability: >85%

Culture morphology:

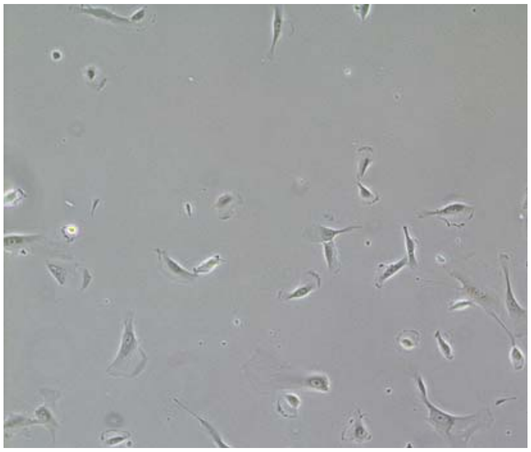

<50% Confluence 100X

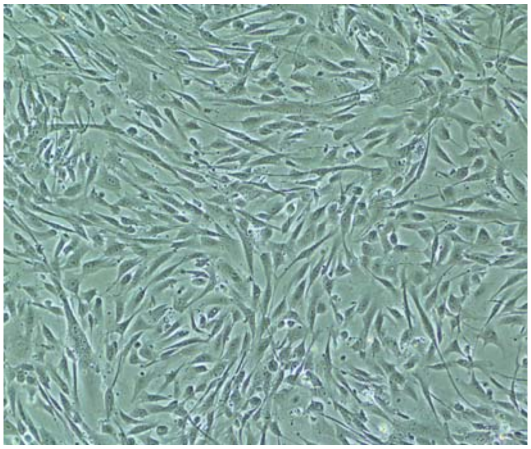

>85% Confluence 100X

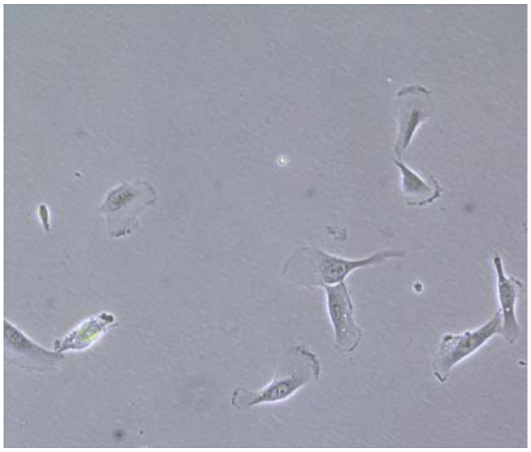

<50% Confluence 200X

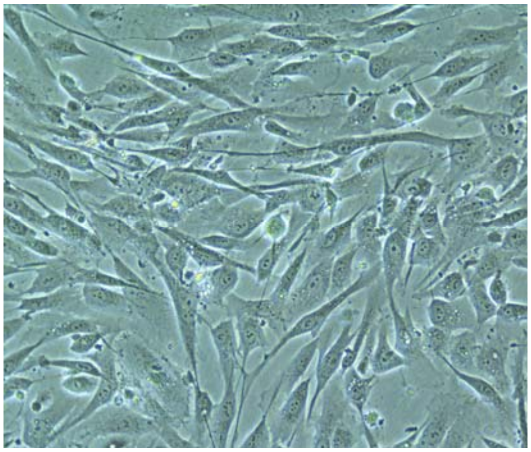

>85% Confluence 200X

Refer to Donor History Record for Additional Donor Details

Samsara Sciences, Inc.
6310 Nancy Ridge Dr., Ste. 106
San Diego, CA 92121
Phone: 858-617-0790
www.samsarasciences.com
info@samsarasciences.com

Know your cells. Trust your data.

Flow cytometry profile

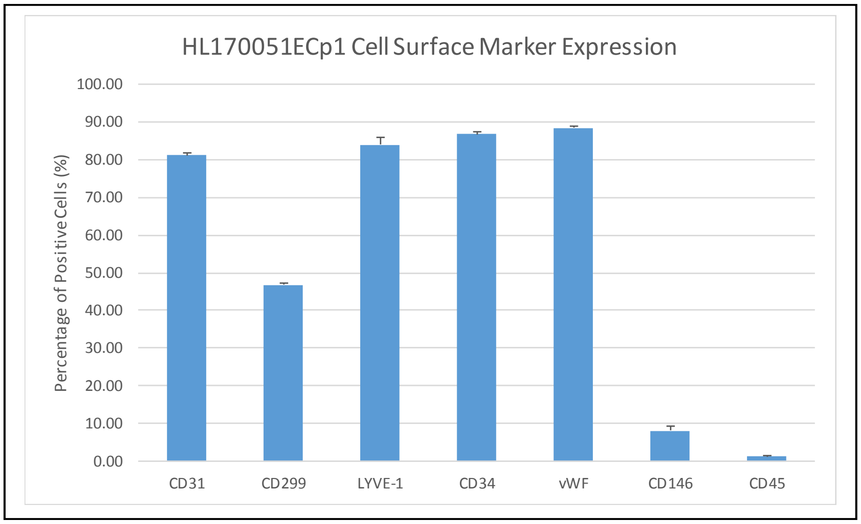

Results above determined by following Samsara endothelial cell care and handling protocols

Samsara Sciences, Inc.
6310 Nancy Ridge Dr., Ste. 106
San Diego, CA 92121
Phone: 858-617-0790
www.samsarasciences.com
info@samsarasciences.com

Know your cells. Trust your data.

DONOR HISTORY REPORT HL160034

DONOR CHARACTERISTICS: HL160034	
**AGE:**	57
**GENDER:**	F
**ETHNICITY:**	African American
**HEIGHT (inches):**	67
**WEIGHT (kg):**	101.9
**BMI:**	35.2
**HbA1c (%):**	Not reported
**CAUSE OF DEATH:**	Cardiac arrest/Anoxia

DONOR MEDICAL HISTORY: HL160034	
**CIGARETTE USE:**	No
**HEAVY ALCOHOL USE (2+ drinks/day):**	No
**IV DRUG USE:**	No
**POSITIVE TOXICOLOGY:**	No
**SEROLOGY:**	EBV+ andCMV+<br>non-reactive for: HIV, HTLV, HBV, HCV, and syphilis
**POSITIVE (BACTERIAL):**	Not reported
**MEDICAL EXAM NOTES:**	None provided
**MEDICATIONS (REGULAR):**	None reported
**PAST MEDICAL HISTORY:**	Sarcoidosis, GERD, Hypertension, hyperlipidemia, depression

PATHOLOGY ASSESSMENT	
**PATHOLOGIST:**	M. Hosseini (UCSD)
**STEATOSIS GRADE:**	0 of 3
**INFLAMMATION SCORE*:**	0-1 of 4
**FIBROSIS SCORE*:**	0 of 4
**NAFLD ACTIVITY SCORE**:**	0 of 8
**OTHER NOTES:**	Focal plasmacytic infiltrate with rare necrosis.

*Batts-Ludwig methodology; **NASH CRN scoring system (Hepatology 2005; 41:1313-1321)

H&E (40x)

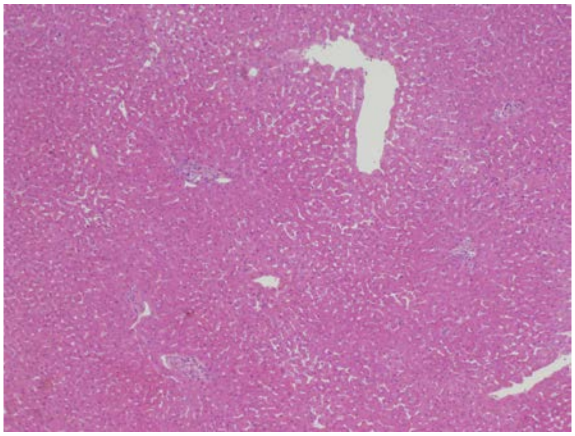

H&E (100x)

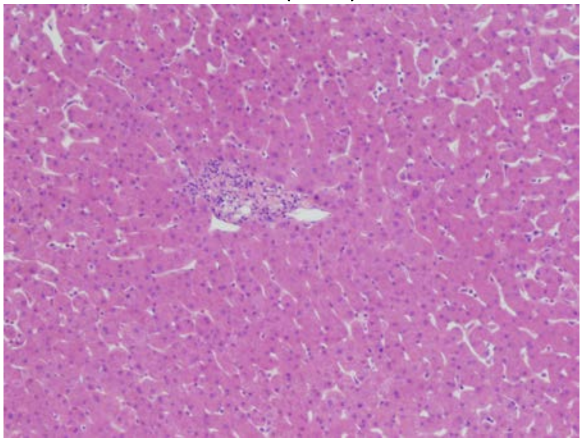

Trichrome (100x)

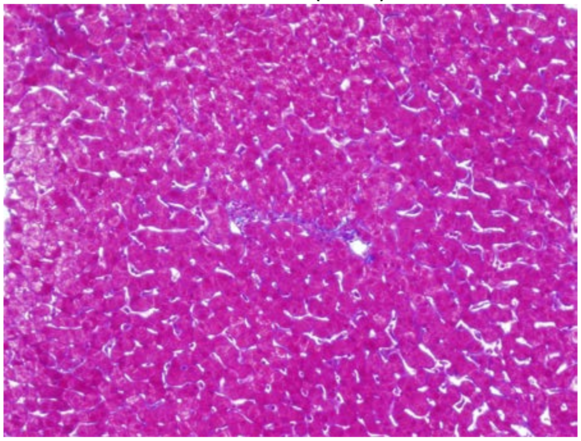

H&E (200x)

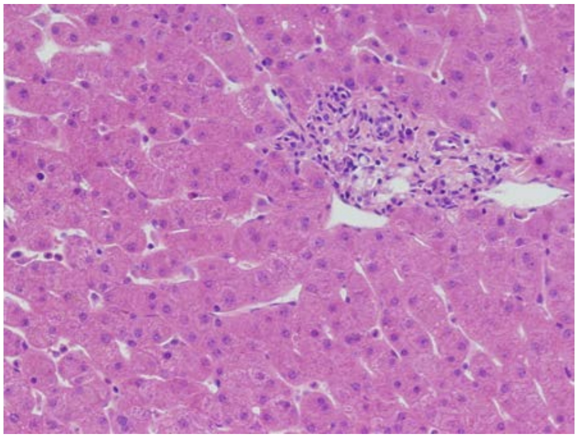

DONOR HISTORY REPORT HL170051

DONOR CHARACTERISTICS: HL170051	
**AGE:**	64
**GENDER:**	M
**ETHNICITY:**	Caucasian
**HEIGHT (inches):**	70
**WEIGHT (kg):**	118
**BMI:**	37.2
**HbA1c (%):**	Not reported
**CAUSE OF DEATH:**	Anoxia/Cardiac arrest

DONOR MEDICAL HISTORY: HL170051	
**CIGARETTE USE:**	No
**HEAVY ALCOHOL USE (2+ drinks/day):**	No
**IV DRUG USE:**	No
**POSITIVE TOXICOLOGY:**	EtOH <9mg/DL
**SEROLOGY:**	CMV+, EBV+<br>Non-reactive for: HIV, HTLV, HBV, HCV and syphilis
**POSITIVE (BACTERIAL):**	Negative
**MEDICAL EXAM NOTES:**	Nothing remarkable
**MEDICATIONS (REGULAR):**	Unknown for depression and acid reflux
**PAST MEDICAL HISTORY:**	Orchidectomy of a cancerous testicle (2002),

PATHOLOGY ASSESSMENT	
**PATHOLOGIST:**	M. Hosseini (UCSD)
**STEATOSIS GRADE:**	0 of 3
**INFLAMMATION SCORE*:**	1 of 4
**FIBROSIS SCORE*:**	0 of 4
**NAFLD ACTIVITY SCORE**:**	1 of 8
**OTHER NOTES:**	Minimal portal inflammation

*Batts-Ludwig methodology; **NASH CRN scoring system (Hepatology 2005; 41:1313-1321)

H&E (50X)

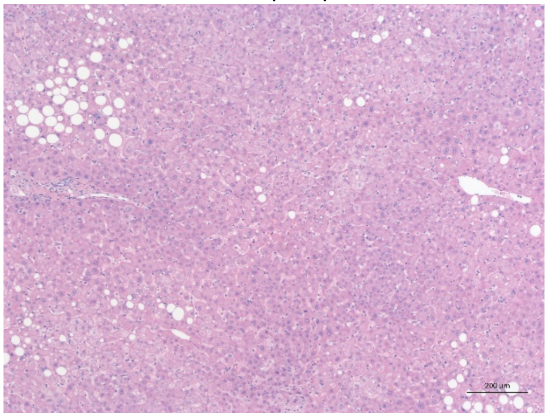

H&E (100X)

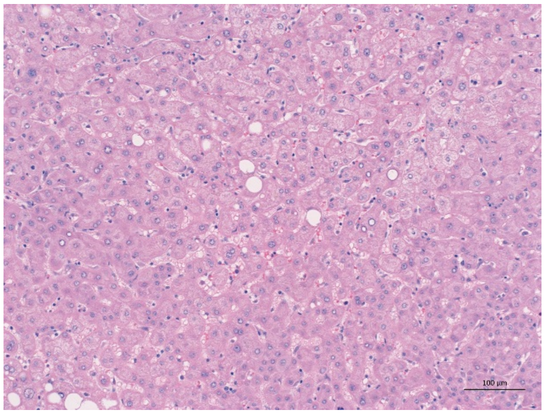

Trichrome (100X)

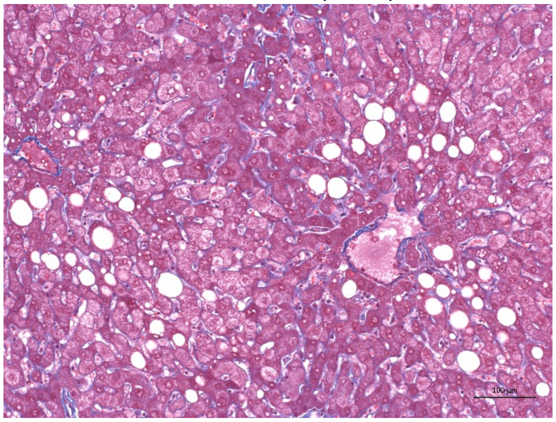

H&E (200X)

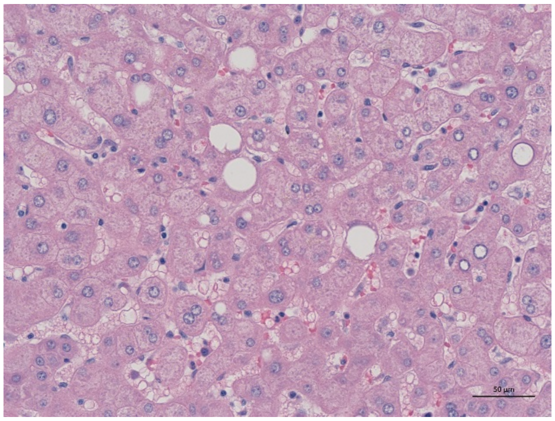

DONOR HISTORY REPORT HL170063

DONOR CHARACTERISTICS: HL170063	
**AGE:**	59
**GENDER:**	F
**ETHNICITY:**	Caucasian
**HEIGHT (inches):**	60
**WEIGHT (kg):**	63.6
**BMI:**	27.4
**HbA1c (%):**	Not done
**CAUSE OF DEATH:**	ICH/Stroke

DONOR MEDICAL HISTORY: HL170063	
**CIGARETTE USE:**	No
**HEAVY ALCOHOL USE (2+ drinks/day):**	No
**IV DRUG USE:**	No
**POSITIVE TOXICOLOGY:**	Negative
**SEROLOGY:**	EBV+<br>Non-reactive for: HIV, HCV, HBV, CMV, Syphilis
**POSITIVE (BACTERIAL):**	No
**MEDICAL EXAM NOTES:**	Not remarkable
**MEDICATIONS (REGULAR):**	None listed
**PAST MEDICAL HISTORY:**	Hyperlipidemia

PATHOLOGY ASSESSMENT	
**PATHOLOGIST:**	Mojgan Hosseini (UCSD)
**STEATOSIS GRADE:**	0 of 3
**INFLAMMATION SCORE*:**	0 of 4
**FIBROSIS SCORE*:**	0 of 4
**NAFLD ACTIVITY SCORE**:**	0 of 8
**OTHER NOTES:**	A little ischemic looking, maybe due to drug injury but minimal.

*Batts-Ludwig methodology; **NASH CRN scoring system (Hepatology 2005; 41:1313-1321)

H&E (50x)

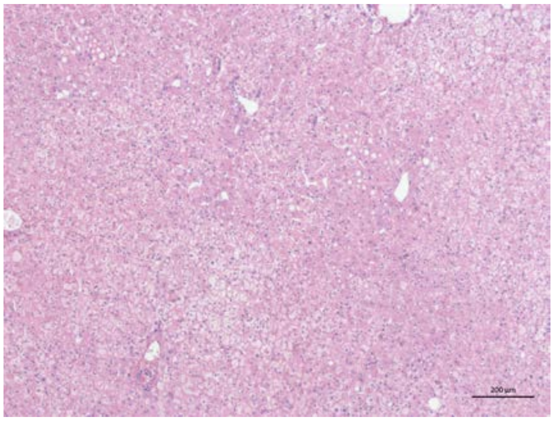

H&E (100x)

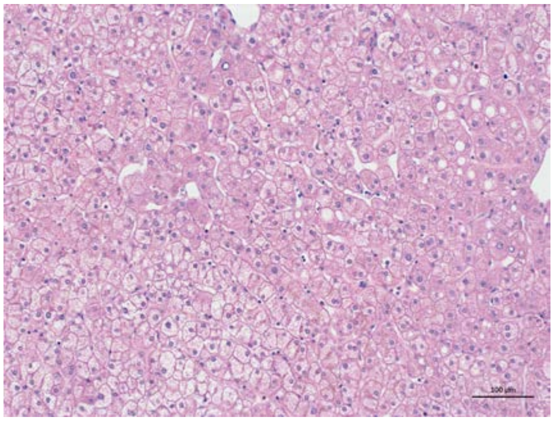

Trichrome (100x)

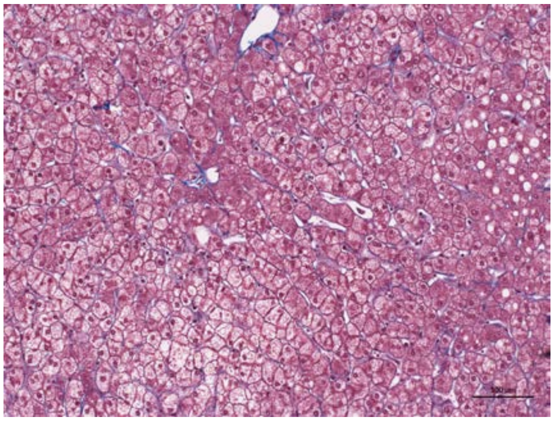

H&E (200x)

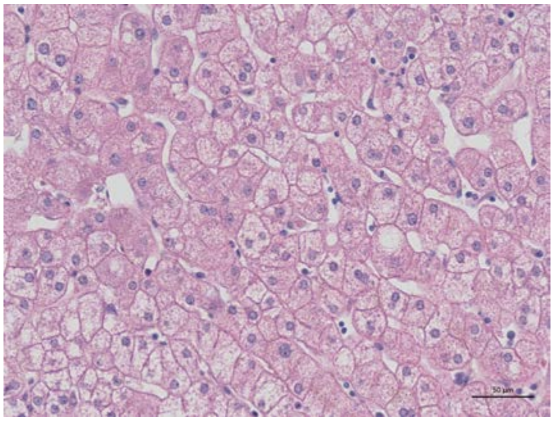

Supplement: Supplementary file 1 — Appendix [file MSB-18-e10947-s009.pdf]
