# Supplementary material for: Dynamic human liver proteome atlas reveals functional insights into disease pathways
Source: Mol Syst Biol. 2022 May 17;18(5):e10947. doi: 10.15252/msb.202210947 (PMC9112488; doi:10.15252/msb.202210947)
Supplement: Supplementary file 2 — Expanded View Figures PDF [file MSB-18-e10947-s015.pdf]

Expanded View Figures

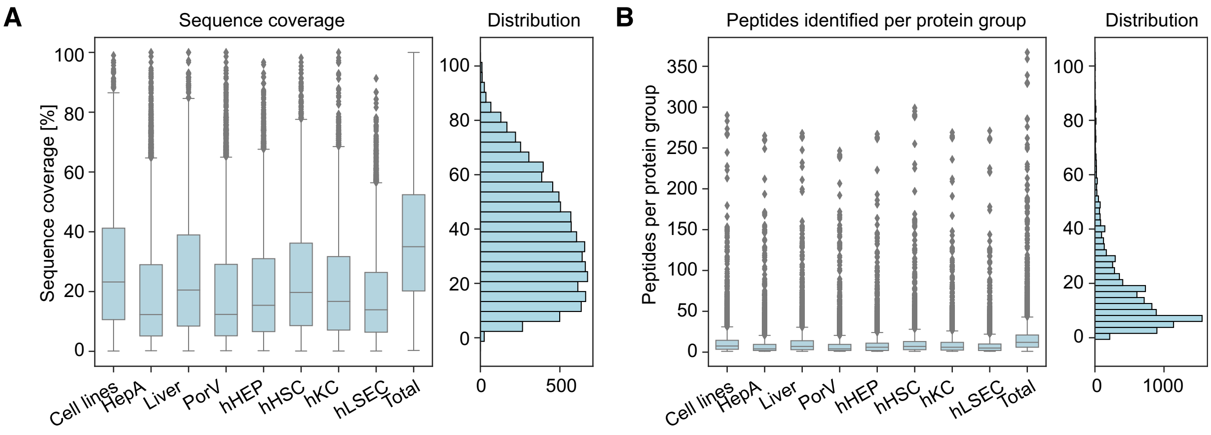

Figure EV1. Proteomics data overview.

A Box plots of sequence coverage distribution of all proteins quantified in each tissue/cell type, and in all samples combined (hHSC: hepatic stellate cell, hHEP: hepatocyte, hKC: Kupffer cell, hLSEC: liver sinusoidal endothelial cell, liver: bulk liver biopsy, HepA: hepatic artery, PorV: portal vein, Cell lines: mixture of human liver-derived immortalized cell lines). Number of independent biological replicates is $n = 4$ for cell lines; $n = 6$ for HepA, Liver and PorV; and $n = 3$ for hHEP, hHSC, hKC and hLSEC. The gray line in the middle of the box is the median, the top and the bottom of the box represent the upper and lower quartile values of the data and the whiskers represent the upper and lower limits for considering outliers (Q3+1.5*IQR, Q1−1.5*IQR) where IQR is the interquartile range (Q3–Q1).

B Box plots of number of identified peptides per protein group in each tissue/cell type, and in all samples combined. Abbreviations and number of independent biological replicates are the same as Panel (A). The gray line in the middle of the box is the median, the top and the bottom of the box represent the upper and lower quartile values of the data and the whiskers represent the upper and lower limits for considering outliers (Q3+1.5*IQR, Q1−1.5*IQR) where IQR is the interquartile range (Q3–Q1).

© 2022 The Authors

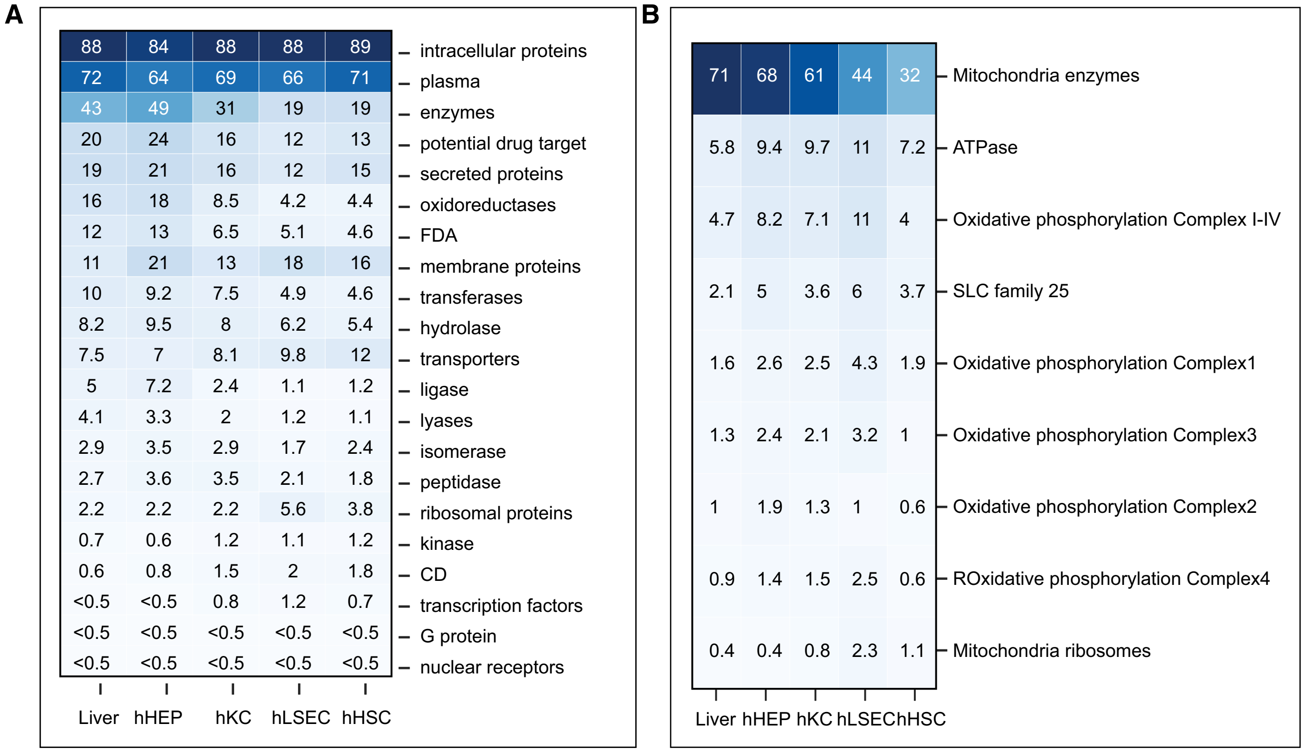

Figure EV2. Cellular mass composition based on protein classes.

A Percent protein mass by protein classes. Protein class annotation was based on the HPA classification.

B Percent protein mass in the mitochondria by selected protein classes. Oxidative phosphorylation is the sum of Complex 1–4.

 © 2022 The Authors

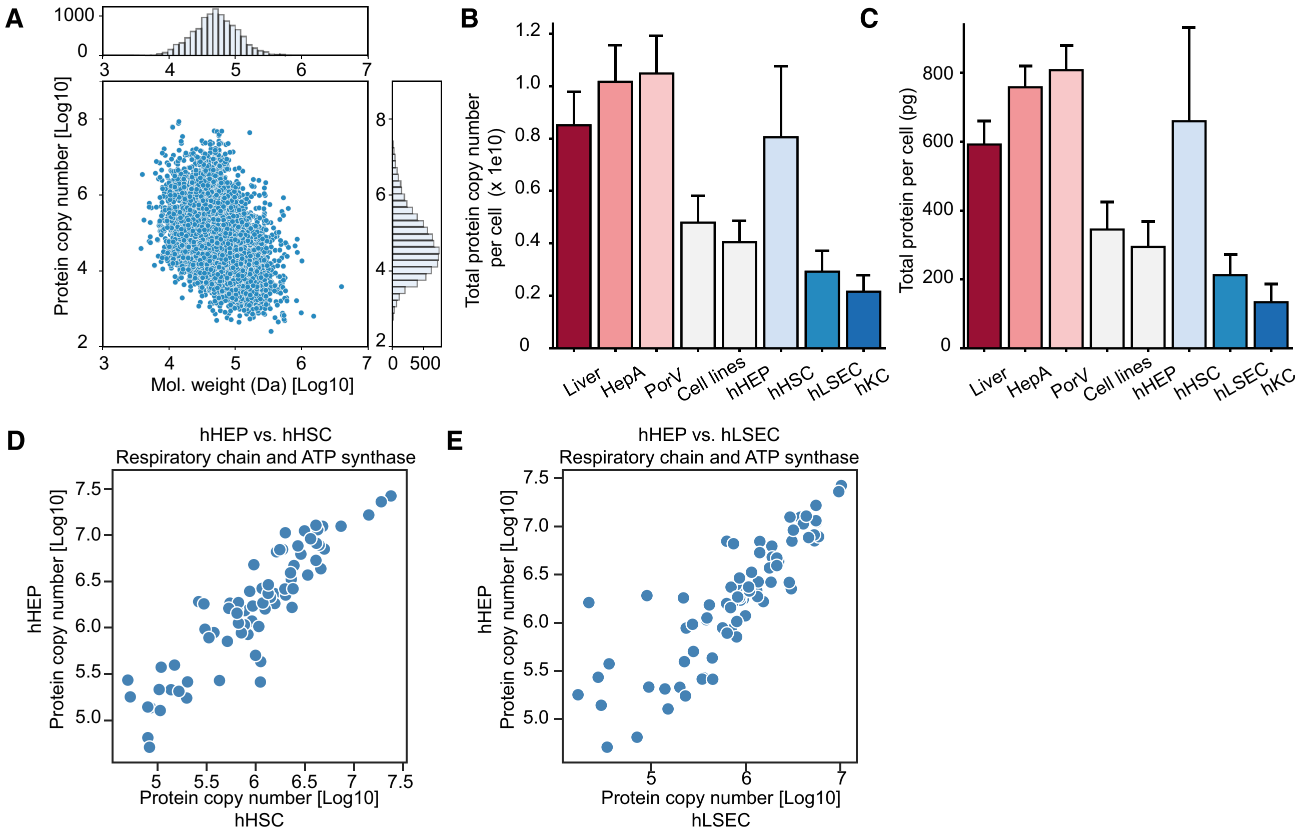

Figure EV3. Protein copy number estimation.

A Averaged protein copy number versus molecular weight in the hepatocyte proteome, with histogram on the *x*- and *y*-axis representing distribution of the molecular weights and protein copy numbers, respectively. Averaged protein copy numbers are derived from $n = 3$ independent biological samples.

B, C Total protein copy number (B) and total protein mass per cell (C) in liver tissues and cell types estimated by the "proteome ruler" approach. Number of biological replicates is $n = 6$ for Liver, HepA, PorV; $n = 4$ for Cell lines and $n = 3$ for hHEP, hHSC, hLSEC and hKC. Values are presented as mean ± s.d.

D, E Protein copy number for members of the oxidative phosphorylation complex I–V in hepatocytes and hepatic stellate cells (D), sinusoidal endothelial cells (E).

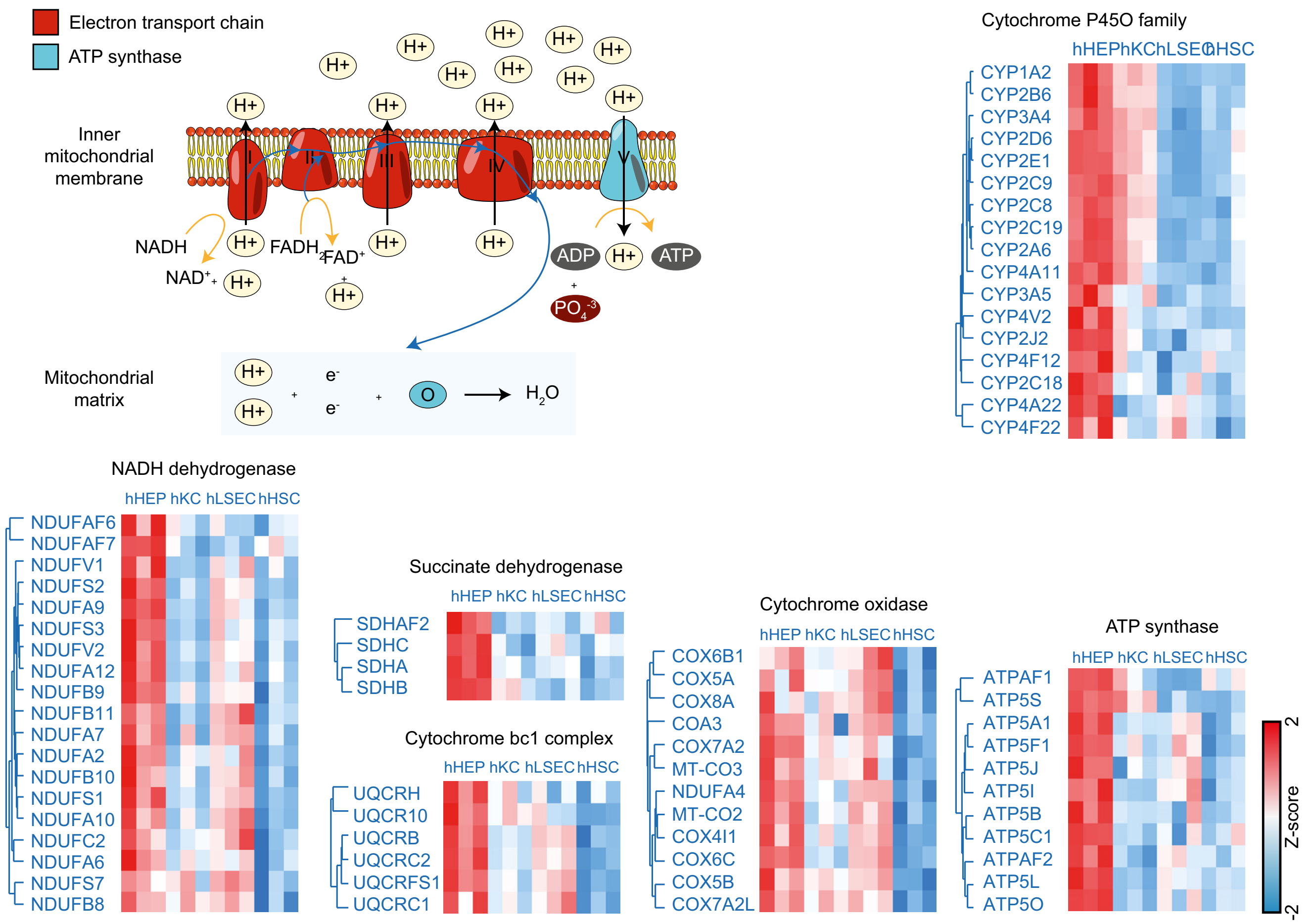

Figure EV4. Hepatocyte-enriched proteins in respiratory chain and drug metabolism.

Clustering of proteins significantly differentially abundant across the four cell types that belong to the electron transport chain and ATP synthase. Values are presented as mean protein abundance followed by *Z*-score normalization across cell types ($n = 3$ independent biological replicates for each cell type).

 © 2022 The Authors

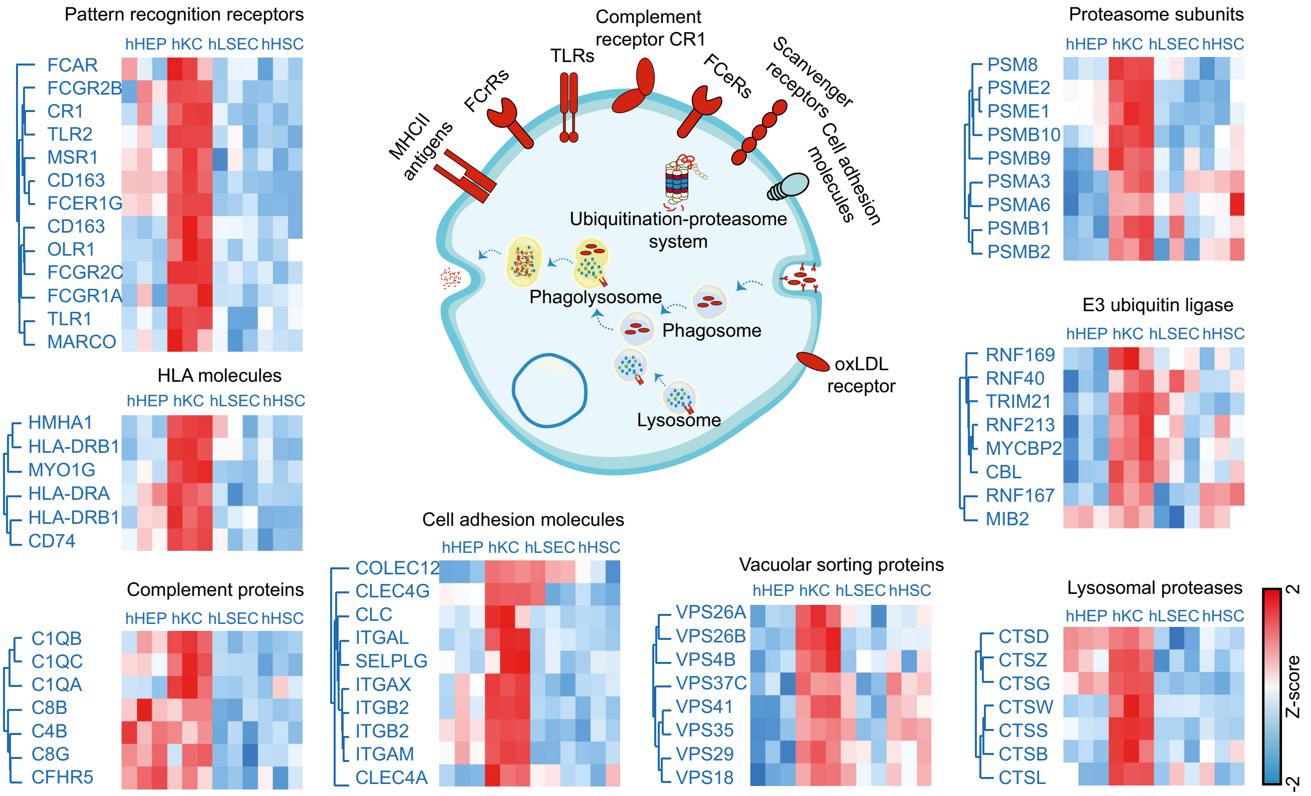

Figure EV5. Kupffer cell-enriched proteins in antigen processing and presentation pathway.

Clustering of proteins significantly differentially abundant across the four cell types that are related to the immune response and protein degradation. Values are presented as mean protein abundance followed by *Z*-score normalization across cell types (*n* = 3 independent biological replicates for each cell type).

© 2022 The Authors

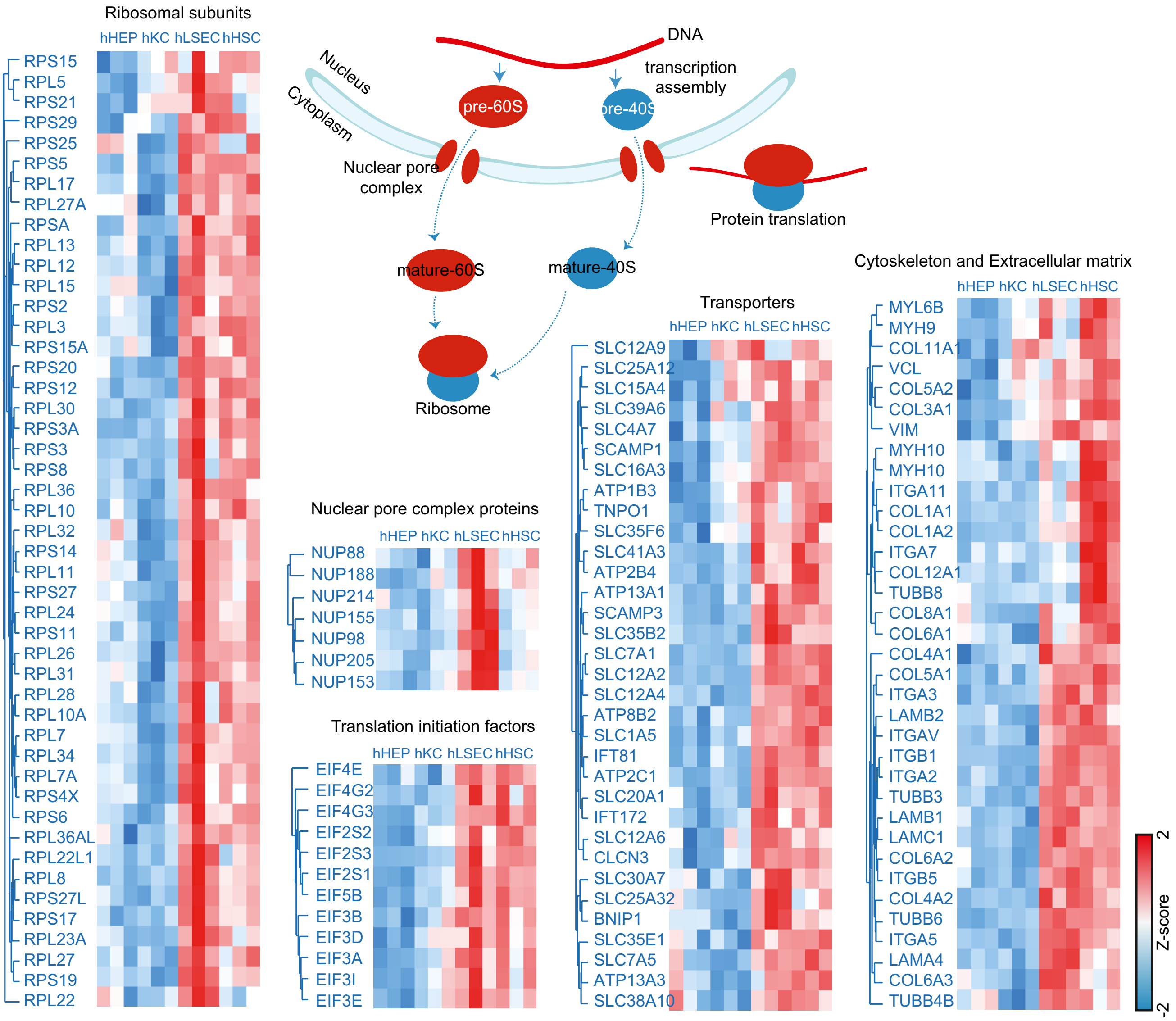

Figure EV6. LSEC-enriched proteins in ribosomal biogenesis and translation and HSC-enriched proteins in cytoskeleton and extracellular matrix.

Clustering of proteins significantly differentially abundant across the four cell types that are involved in ribosomal biogenesis and translation, as well as cytoskeleton and extracellular matrix. Values are presented as mean protein abundance followed by *Z*-score normalization across cell types ($n = 3$ independent biological replicates for each cell type).

 © 2022 The Authors
