# Supplementary material for: Dynamic human liver proteome atlas reveals functional insights into disease pathways
Source: Mol Syst Biol. 2022 May 17;18(5):e10947. doi: 10.15252/msb.202210947 (PMC9112488; doi:10.15252/msb.202210947)
Supplement: Supplementary file 16 [file MSB-18-e10947-s012.pdf]

molecular systems biology

# Dynamic human liver proteome atlas reveals functional insights into disease pathways

Matthias Mann, Lili Niu, Philipp Geyer, Rajat Gupta, Alberto Santos, Florian Meier, Sophia Doll, Nicolai Jakob Wewer Albrechtsen, Sabine Klein, Cristina Ortiz, Frank Uschner, Robert Schierwagen, and Jonel Trebicka
**DOI: 10.15252/msb.202210947**

*Corresponding author(s): Matthias Mann (mmann@biochem.mpg.de) , Jonel Trebicka (Jonel.Trebicka@kgu.de)*



## Review Timeline:

| | |
|---|---|
| Submission Date: | 28th Jan 22 |
| Editorial Decision: | 25th Mar 22 |
| Revision Received: | 14th Apr 22 |
| Editorial Decision: | 27th Apr 22 |
| Revision Received: | 27th Apr 22 |
| Accepted: | 28th Apr 22 |

*Editor: Maria Polychronidou*


## Transaction Report:

(Note: With the exception of the correction of typographical or spelling errors that could be a source of ambiguity, letters and reports are not edited. Depending on transfer agreements, referee reports obtained elsewhere may or may not be included in this compilation. Referee reports are anonymous unless the Referee chooses to sign their reports.)

Thank you again for submitting your work to Molecular Systems Biology. I would like to apologise for the delay in getting back to you, which was due to the fact that after repeated reminders we still have not received the comments of reviewer #1. To not delay the process any further, and given that the recommendations of reviewers #2 and #3 are similar, we have now decided to proceed with making a decision based on the two available reports. The two reviewers acknowledge that the study presents a valuable resource and they are supportive. However, they raise a series of concerns, which we would ask you to address in a revision.

The comments of the referees are mostly minor and straightforward to address so I think there is no need to repeat any of them here. In line with the comment of reviewer #1 we would ask you to include a link to the web app in the manuscript (and in the abstract) and to ensure that the Github link is functional.

All issues raised by the referees would need to be satisfactorily addressed. Please let me know in case you would like to discuss in further detail any of the issues raised.

On a more editorial level, we would ask you to address the following points:

REFEREE REPORTS
--------------------------------------------------------------------------

Reviewer #2:

Niu et al present a large resource of liver proteomics ranging from a deep atlas of cell lines, primary cells, and human material based on DDA fraction analysis, followed up by quantitive assessment in DIA mode of a primary cell adaptation time course and liver disease patient cohort. There is a fairly deep comparison of different cell lineages which I think leads to very interesting conclusions. The copy number analysis also adds value gives a more quantitative perspective to their atlas which is welcome. The insights into proteins that are unique or characteristic to given lineages is very worthwhile. My view is that the methodology is sound, the data quality is high, the presentation is clear, and the conclusions drawn are reasonable and supported by the data. Overall, my view is that this is a very strong piece of work that will add substantial value to the literature.

I am happy to say I find very little to complain about in this study. The only minor suggestions I have are:

1. The authors make much of their web app for visualization. This is of course very welcome but unfortunately I cannot see a link for this resource. If they want to present this as a major component of the paper then the reviewers need to be able to see this. Similarly, the authors point to a github repo for the analysis code associated with the paper but the link does not work so I assume this has not been made public? This should also be made available for reviewers.

2. The presentation style seems (to my eye) somewhat verbose. I think the text would have more impact if presented more concisely. I understand that this is to some extent a matter of opinion so not much turns on this comment and I think the editor (or other reviewers) could also contribute an opinion here.

Reviewer #3:

The authors describe an understanding of the liver proteome atlas using LC/MS-based proteomics. They performed a deep proteome analysis using several liver-based tissues and samples and describe in detail the molecular functions and biological pathways. Finally, the authors build a web-based dashboard application for data exploration. However, the authors should address some minor comments in random order of importance before acceptance of the publication.

Minor comments:
1. The authors should provide some details about the method of imputation for the PCA and if there was a pre-filtering based on the percentage of valid values.
2. Figure 1d: Please define the terms in the legend.
3. Figure 1c: Would be interesting to describe the top 50 most abundant proteins.
4. Figure 2a: Please define the nature of the 86 proteins uniquely identified in HepA + PorV.
5. Figure 2b: some blood-related proteins belong to the most abundant proteins (e.g, albumin, hemoglobin). How to address this potential 'contamination'?
6. Figure 2c/d: surprised that the blood proteins do not contribute to the separation between the cell lines and the biopsy tissues. Might be helpful to perform correlation analysis because imputation might induce a bias in the results.
7. Figure 2d: What are the ranks for the proteins highlighted in blue, red, and black? Is the separation driven the most/low abundant proteins?
8. Figure 3a: Out of curiosity, can the uniquely identified proteins in each of the cell types be considered as 'cell-type' specific? Might be interested to perform a Fisher test.
9. Figure 3d: hKC seem to be similar as macrophages. Do hKC have similar protein rank order than macrophages?
10. Figure 5: Out of curiosity, what are the proteins/pathways corresponding to the non-significant proteins?
11. Figure 6: The authors should indicate the reason of using these three particular time points (why not every day for one week or adding day14?).
12. Figure 6: Why did the authors perform DIA analysis without using their previously developed spectral library?
13. Figure 6: Any particular reason why the number of proteins in hHEP keeps increasing while the other two are relatively stable?
14. Figure 6: What are the common proteins between the three cell types that changed over time?
15. Figure 6f: Do they authors observe any significant decrease of protein intensity?
16. Figure 7: Reasonable number of samples in the cohort. Any confounder(s) regressed in the ANOVA (e.g., age, sex, 'obesity')?

17. Figure 7: The authors wrote: "These results provide the molecular basis for the clinical observations of liver cirrhosis, characterized by the replacement of normal liver tissue by scar tissue, the formation of new vessels leading to abnormal angioarchitecture in the cirrhotic liver, and the compromised immune system and dysregulated immune cell activation (Schierwagen et al, 2020; Trebicka et al, 2019)."
Did they observe an increase of the blood-related proteins? or proteins that might be considered as "specific to HepA or PorV"?

**Point by point answers to reviewers for 'Dynamic human liver proteome atlas reveals functional insights into disease pathways' by Lili Niu et al.**

**Reviewer #2:**

Niu et al present a large resource of liver proteomics ranging from a deep atlas of cell lines, primary cells, and human material based on DDA fraction analysis, followed up by quantitive assessment in DIA mode of a primary cell adaptation time course and liver disease patient cohort. There is a fairly deep comparison of different cell lineages which I think leads to very interesting conclusions. The copy number analysis also adds value gives a more quantitative perspective to their atlas which is welcome. The insights into proteins that are unique or characteristic to given lineages is very worthwhile. My view is that the methodology is sound, the data quality is high, the presentation is clear, and the conclusions drawn are reasonable and supported by the data. Overall, my view is that this is a very strong piece of work that will add substantial value to the literature.

I am happy to say I find very little to complain about in this study.

Authors' response: We thank the reviewer for this very positive and kind consideration of our work and also for the suggestions which we addressed in the point-by-point answers below.

The only minor suggestions I have are:

1. The authors make much of their web app for visualization. This is of course very welcome but unfortunately I cannot see a link for this resource. If they want to present this as a major component of the paper then the reviewers need to be able to see this. Similarly, the authors point to a GitHub repo for the analysis code associated with the paper but the link does not work so I assume this has not been made public? This should also be made available for reviewers.

We apologize for this inconvenience, which was caused by us not making the GitHub repository publicly available. We have now made the web-based dashboard application public at www.liverproteome.org. We also made the GitHub repository public at https://github.com/llniu/Human_Liver_Proteome.

2. The presentation style seems (to my eye) somewhat verbose. I think the text would have more impact if presented more concisely. I understand that this is to some extent a matter of opinion so not much turns on this comment and I think the editor (or other reviewers) could also contribute an opinion here.

We thank you for this comment. In the revised manuscript, we tried to rephrase whatever seemed to be lengthy and hope that it is more concise now. Mainly we have tightened the introduction but also elsewhere throughout the manuscript.

**Reviewer #3:**

The authors describe an understanding of the liver proteome atlas using LC/MS-based proteomics. They performed a deep proteome analysis using several liver-based tissues and samples and describe in detail the molecular functions and biological pathways. Finally, the authors build a web-based dashboard application for data exploration. However, the authors should address some minor comments in random order of importance before acceptance of the publication.

Authors' response: Thank you for the positive and detailed consideration of our work. Inspired by your comments we have performed additional analyses that uncovered more biological insights, strengthening our study. We address the comments in the point-by-point answers below.

Minor comments:

1. The authors should provide some details about the method of imputation for the PCA and if there was a pre-filtering based on the percentage of valid values.

We have now included details about whether data was pre-filtered and the method of imputation for the PCA. This is on Page 22, Line 821-824 in the revised manuscript with track changes (line 808-811 clean version), also pasted below.

*"For the PCA, no pre-filtering was applied due to the diversity of sample types and relatively small number of biological replicates. Missing values were imputed by drawing from a down-shifted normal distribution relative to that of a sample's proteome abundance distribution (down-shifted mean by 1.8 standard deviation (SD) and scaled SD (0.3)). The same imputation method was applied throughput all analyses."*

2. Figure 1d: Please define the terms in the legend.

We agree with the referee and have now defined the terms in figure 1A-D in the legend on Page 30, Line 1144-1161 (Line 1130-1144 clean version).

"***A.*** *Overview of biological material used for generating the liver proteome atlas. (hHSC: hepatic stellate cell, hHEP: hepatocyte, hKC: Kupffer cell, hLSEC: liver sinusoidal endothelial cell; TWNT4 and LX2: immortalized human hepatic stellate cell line, SK-Hep-1: human hepatic adenocarcinoma cell line, HepG2: human liver cancer cell line). Number of biological replicates is n=6 for bulk liver, hepatic artery and portal vein; n=3 for hHEP, hLSEC, hHSC, hKC and n=1 for HepG2, SK-Hep1, LX2 and TWNT4. No additional replications of the experiment was done in laboratory.*

***B.*** *Total quantified proteome depth in tissues (n=18), primary cells (n=12), immortalized cell lines (n=4) and all samples (n=34). In all cases, n means biological replicates unless otherwise indicated. The upper- and lower panel shows the number of quantified protein groups and peptides, respectively.*

***C.*** *Dynamic range of the different proteomes based on median LFQ intensity ordered by abundance rank (Liver: bulk liver biopsy, HepA: hepatic artery, PorV: portal vein, Cell lines: mixture of human liver-derived immortalized cell lines). Number of biological replicates is same as Panel (A).*

***D.*** *Principal component analysis (PCA) of all proteomes based on their proteome profiles. For abbreviations please refer to Panel (A).* ***e.*** *KEGG pathway coverage in this dataset, with major metabolic pathways highlighted.*

"

3. Figure 1c: Would be interesting to describe the top 50 most abundant proteins.

Thank you for this comment. In Figure 1c, we aim to show the differences in dynamic range of protein abundance between the different tissue- and cell types. In order to not overload the figure, we described the top 10 most abundant proteins in Figure 2c and Figure 3d in detail. We hope the reviewer find this sufficient, otherwise we would be happy to add the top 50 most abundant proteins in a separate supplementary figure.

4. Figure 2a: Please define the nature of the 86 proteins uniquely identified in HepA + PorV.

These proteins characterize the unique muscle-fiber structure of blood vessels and tend to be present in moderate to low abundance and most of them are typically not covered by standard single-run tissue proteomics workflows. There are only 12 unique proteins to HepA or PorV among the 3000 most abundant proteins. In total 24 of the 86 unique proteins are within the top 5000. Ontology enrichment analysis on these proteins using the compartments.jensenlab.org database identified the following cellular compartments as significantly enriched: "myofibril and Contractile fiber" (MYH7B, TPM1, ANKRD1, SORBS2, ANKRD2, SYNE1, PLEC), "median body" (SFRP4, CTF1, CA5B, CYS1), perineuronal net (VCAN, NME4, CTNNA3, CHST3) and extracellular matrix (POSTN, VCAN, ECM2, SFRP2, WNT11, FN1, PLEC). We have now described the nature of these 86 proteins on Page 5, line 158-162 (line 148-152 clean version).

"*The 86 proteins are characteristic of the unique muscle-fiber structure of blood vessels, such as Myosin-7B, Tropomyosin alpha-1 chain and Plectin. They tend to be present in moderate to low abundance and most of them are typically not covered by standard single-run tissue proteomics workflows. To illustrate, there are only 12 unique proteins to HepA or PorV among the 3000 most abundant proteins, and 24 among the top 5000.* "

5. Figure 2b: some blood-related proteins belong to the most abundant proteins (e.g, albumin, hemoglobin). How to address this potential 'contamination'?

We agree with the reviewer that this is an important concern. Although we flushed and rinsed the liver biopsy, hepatic artery and portal vein upon sampling, we cannot exclude the possibility of some blood contamination. Liver perfusion would be a good way to remove blood contamination, however, it is only practical in mouse models. In the revised version, we have highlighted proteins in Figure 2b that may originate from blood contamination on Page 5, line 171-173 (line 161-163 clean version), and stated this as a limitation on Page 16, line 659-662 (line 653-656 clean version).

"*The high abundance of blood related proteins (ALB, HBB and HBA1) could be due to some blood contamination, although the samples used in the analysis were flushed and rinsed upon sampling*."

"*A limitation to this study is that we cannot exclude the possibility of some blood contamination in the sampling of liver biopsies, hepatic artery and portal vein even though we rinsed and flushed the hepatic vessels upon sampling. Liver perfusion would be a good way to remove blood contamination, however, it is only practical in mouse models*."

6. Figure 2c/d: surprised that the blood proteins do not contribute to the separation between the cell lines and the biopsy tissues. Might be helpful to perform correlation analysis because imputation might induce a bias in the results.

We have now highlighted the most abundant blood proteins (colored in red) on the loading plot of the PCA, and they do contribute to the separation between cell lines and tissues. This reflects the fact that they are highly abundant in the biopsies and the blood vessels compared to the cell lines. However, our goal was to look for proteins that separate blood vessels, liver biopsies and the cell lines, respectively from the rest, so

these blood proteins were not highlighted in the article. If the figure below does not fully address the reviewer's concern, we would be happy to revisit this.

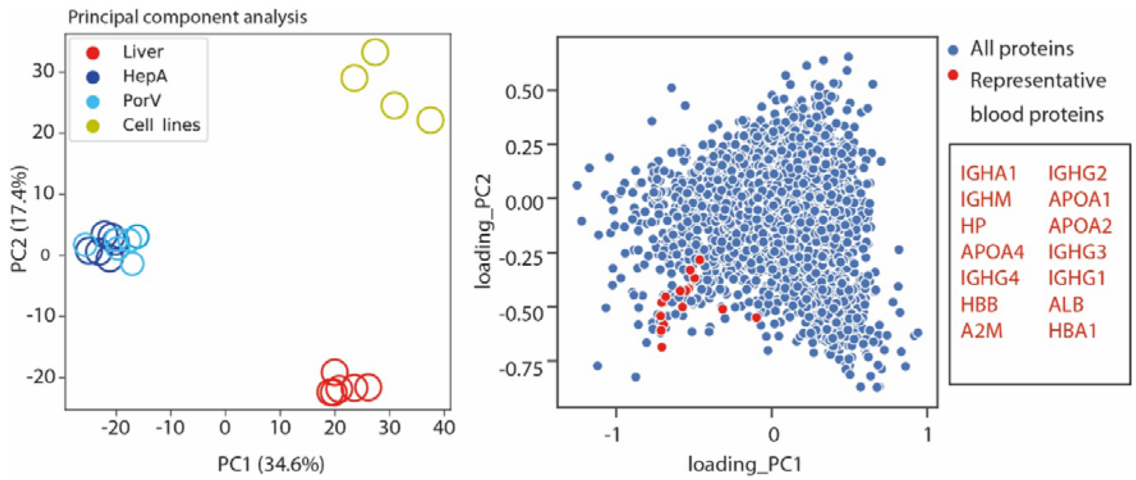


7. Figure 2d: What are the ranks for the proteins highlighted in blue, red, and black? Is the separation driven the most/low abundant proteins?

We calculated the abundance rank of proteins highlighted in Figure 2d. For liver biopsies and the blood vessels (HepA and PorV), it is a mix of most- and low abundant proteins driving their separation (Revision Table 1 below), while majority of them are most abundant ones. For cell lines, the proteins are moderately abundant.

Revision Table 1: Abundance rank of proteins highlighted in Figure 2d.

| Protein ID | Gene name | vessels log10 intensity | cell lines log10 intensity | liver log10 intensity | rank in vessels | rank in cell lines | rank in liver |
|---|---|---|---|---|---|---|---|
| P05181 | CYP2E1 | 8.59 | 8.20 | 10.24 | 2619 | 4892 | **188** |
| Q9H2A2 | ALDH8A1 | 7.74 | 7.62 | 9.91 | 5856 | 7182 | **372** |
| E7ENE7 | CYP2D6 | 8.13 | 7.76 | 9.90 | 4371 | 6623 | **382** |
| P22310 | UGT1A4 | 7.36 | 7.41 | 9.89 | 7126 | 7911 | **388** |
| Q02928 | CYP4A11 | 7.31 | 7.82 | 9.82 | 7521 | 6416 | **458** |
| Q08477-2 | CYP4F3 | 7.16 | 7.82 | 9.80 | 9808 | 6420 | **488** |
| P11226 | MBL2 | 7.92 | 7.73 | 9.63 | 5184 | 6774 | **705** |
| Q9H2M3 | BHMT2 | 7.16 | 7.32 | 9.62 | 9812 | 8200 | **707** |
| O75452 | RDH16 | 7.14 | 7.17 | 9.55 | 10034 | 8861 | **816** |
| Q6NWU0 | CYP2D6 | 7.20 | 7.17 | 7.65 | 9165 | 8873 | **7000** |
| E7EVL8 | CYP4A11 | 7.13 | 7.26 | 7.47 | 10148 | 8432 | **7575** |
| Q08477 | CYP4F3 | 7.19 | 7.08 | 7.13 | 9341 | 9366 | **9163** |
| A0A1B0GU72 | CYP2D6 | 7.11 | 6.94 | 7.07 | 10261 | 10132 | **9652** |
| P35749 | MYH11 | 11.82 | 9.33 | 10.16 | **4** | 1125 | 227 |
| P17661 | DES | 11.35 | 8.96 | 9.35 | **14** | 2089 | 1166 |
| P51911 | CNN1 | 11.23 | 8.31 | 9.24 | **19** | 4440 | 1374 |
| P20774 | OGN | 11.08 | 8.32 | 9.83 | **28** | 4374 | 457 |

| | | | | | | | |
|---|---|---|---|---|---|---|---|
| P51888 | PRELP | 10.98 | 7.38 | 10.16 | **39** | 8006 | 228 |
| P15502-4 | ELN | 10.54 | 7.46 | 9.21 | **84** | 7724 | 1422 |
| P29536 | LMOD1 | 10.23 | 7.85 | 8.63 | **150** | 6326 | 3200 |
| P35749-4 | MYH11 | 9.41 | 6.92 | 6.94 | **621** | 10215 | 10348 |
| F5GZK2 | COL21A1 | 9.33 | 6.57 | 7.37 | **745** | 10526 | 7859 |
| G5E950 | ELN | 8.99 | 6.97 | 7.62 | **1397** | 10022 | 7084 |
| H7C3K0 | ELN | 7.90 | 7.63 | 7.09 | **5290** | 7155 | 9490 |
| P35749-2 | MYH11 | 7.47 | 7.10 | 7.20 | **6639** | 9269 | 8648 |
| E7EQH8 | ELN | 7.32 | 7.14 | 7.08 | **7477** | 9067 | 9562 |
| F8W930 | IGF2BP2 | 7.23 | 9.47 | 7.56 | 8762 | **830** | 7302 |
| Q9NZI8 | IGF2BP1 | 7.03 | 9.31 | 6.98 | 10491 | **1166** | 10230 |
| O00622 | CYR61 | 8.08 | 8.95 | 7.37 | 4579 | **2127** | 7863 |
| Q71DI3 | HIST2H3A | 7.55 | 8.86 | 7.02 | 6433 | **2446** | 10033 |
| P52926 | HMGA2 | 7.20 | 8.76 | 6.94 | 9174 | **2763** | 10353 |
| Q15645 | TRIP13 | 7.37 | 8.56 | 7.34 | 7093 | **3468** | 7946 |
| B3KWK7 | IGFBP3 | 8.60 | 8.02 | 8.82 | 2605 | **5570** | 2542 |

8. Figure 3a: Out of curiosity, can the uniquely identified proteins in each of the cell types be considered as 'cell-type' specific? Might be interested to perform a Fisher test.

Thank you for the very interesting suggestion. These proteins include those that were detected in one, two or three biological replicates of the same liver cell type but not others. It is possible that these proteins were too low abundant to be robustly detected in all biological replicates of the same cell type, or that they were randomly detected in only one of the four cell types. Fisher exact test was significant when a protein was detected in at least two biological replicates of the same cell type (Table 1 below) but not in any other cell type, making 284 proteins "cell-type" specific candidates (59 for hHEP, 107 for hHSC, 91 for hKC and 27 for hLSEC). Despite this, we took a more stringent criteria in defining "cell-type" specific proteins – proteins that were detected in all three biological replicates of the same cell type but not in any other cell types, as shown in Figure 4d. To incorporate the reviewer's comment, we have added Fisher exact test result and its implications on Page 5, line 195-197 in the revised manuscript (line 186-189 clean version).

*"Among the proteins that were uniquely identified in one cell type, 284 were detected in at least two of three biological replicates (Fisher exact test p<0.05, one cell type against the rest), making them candidates for cell-type specific proteins."*

Revision Table 2: Fisher exact test

Situation 1: protein was detected in 1/3 biological replicates of the same cell type but not others

| | Detected | Not detected | Total | Two-tailed p-value |
|---|---|---|---|---|
| Cell type to be tested | **1** | 2 | 3 | |
| Other cell types | 0 | 9 | 9 | 0.25 |
| Total | 1 | 11 | 12 | |

Situation 2: protein was detected in 2/3 biological replicates of the same cell type but not others

| | Detected | Not detected | Total | Two-tailed p-value |
|---|---|---|---|---|

| | | | | |
|---|---|---|---|---|
| Cell type to be tested | **2** | 1 | 3 | **0.045*** |
| Other cell types | 0 | 9 | 9 | |
| Total | 2 | 10 | 12 | |

Situation 3: protein was detected in 3/3 biological replicates of the same cell type but not others

| | Detected | Not detected | Total | Two-tailed p-value |
|---|---|---|---|---|
| Cell type to be tested | **3** | 0 | 3 | **0.0045*** |
| Other cell types | 0 | 9 | 9 | |
| Total | 3 | 9 | 12 | |

* Significance level p<0.05

9. Figure 3d: hKC seem to be similar as macrophages. Do hKC have similar protein rank order than macrophages?

The liver contains two distinct populations of macrophages: monocyte-derived macrophages (MDMs) and the liver-resident Kupffer cells, with the latter dominating the hepatic macrophage pool with as much as 80-90% (Bilzer et al., 2006). While we did not isolate MDMs in our study, it is highly possible that MDMs exhibit a similar proteome profile as Kupffer cells, upon infiltration of the liver. At least they exhibit most similar transcriptome profile to each other as compared to the rest of human liver cell populations (Wang et al., 2021).

10. Figure 5: Out of curiosity, what are the proteins/pathways corresponding to the non-significant proteins?

In total, 4,694 of 8,866 proteins were not significantly differentially abundant across the four liver cell types in ANOVA. Ontology enrichment analysis on these non-significant proteins did not identify significantly enriched biological processes, but a few terms related to metabolic process were significantly depleted (Table below).

Review Table3: Ontology enrichment analysis on non-significant proteins in the differential abundance analysis between four liver cell types.

| GO biological process | In reference # | In test set # | expected # | (+/-) | Fold enrichment | P-value | FDR |
|---|---|---|---|---|---|---|---|
| small molecule metabolic process | 1111 | 446 | 596.67 | - | 0.75 | 1.99E-08 | 6.78E-05 |
| oxoacid metabolic process | 643 | 216 | 345.33 | - | 0.63 | 2.12E-10 | 1.45E-06 |
| carboxylic acid metabolic process | 624 | 209 | 335.13 | - | 0.62 | 3.12E-10 | 1.42E-06 |
| organic acid metabolic process | 653 | 217 | 350.7 | - | 0.62 | 6.26E-11 | 8.56E-07 |
| monocarboxylic acid metabolic process | 382 | 121 | 205.16 | - | 0.59 | 1.05E-07 | 2.40E-04 |
| small molecule catabolic process | 281 | 88 | 150.91 | - | 0.58 | 3.88E-06 | 6.63E-03 |
| organic acid catabolic process | 191 | 45 | 102.58 | - | 0.44 | 8.49E-08 | 2.32E-04 |
| carboxylic acid catabolic process | 187 | 44 | 100.43 | - | 0.44 | 1.32E-07 | 2.58E-04 |

11. Figure 6: The authors should indicate the reason of using these three particular time points (why not every day for one week or adding day14?).

We selected 1-, 3, and 7-day cultures based on a previous study in which we observed substantial proteome remodeling during 7-day culture of primary hepatocytes freshly isolated from mice. Furthermore, HSC and LSEC change the phenotype upon culturing. For these reasons, we did not further extend the culture period to more than one week. This is also because primary cells have limited growth potential. Even with optimal growth conditions, they typically only last several days and eventually die without going for more passages. In addition, due to limited sample availability for primary human hepatic cells, we avoided sampling every day. To incorporate the reviewer's question, we added the reason of using these three time points in the revised manuscript on Page 10, line 409-412 (line 400-403 clean version).

"*We selected the three time points based on a previous study in which we observed substantial proteome remodeling during 7-day culture of primary hepatocytes freshly isolated from mice* (Azimifar et al., 2014)*. We did not further extend the culture period or sample more frequently due to limitations in sample availability and the limited growth potential of primary cells in culture.* "

12. Figure 6: Why did the authors perform DIA analysis without using their previously developed spectral library?

Thank you for raising this point. Ideally, a spectral library should be acquired at the same chromatography and mass spectrometric settings as the single-shot DIA analysis. By the time we were ready to acquire the proteomics data for the primary cell culture experiment, the Q Exactive HF-X Orbitrap, which we used to acquire data to build the spectral library was not available in our laboratory anymore, as we upgraded all instruments to Orbitrap Exploris 480. These Exploris instruments are equipped with the FAIMS Pro Interface, which is a differential ion mobility device that can separate gas-phase ions based on their characteristic differences in mobility in high and low electric field. This device allows the simultaneous analysis of multiple gas-phase fractions by fast internal compensation voltage (CV) stepping in a single run, and can increase the measured proteome depth with similar quantitative reproducibility to that of single-shot experiments without FAIMS (Bekker-Jensen et al., 2020; Hebert et al., 2018). We therefore took advantage of this new feature and acquired data with an optimized acquisition method using DIA mode with intra-analysis CV-switching. Accordingly, we built a new spectral library from seven gas phase fractionations by stepping the CV from -40V to -70V with an increment of -5V.

13. Figure 6: Any particular reason why the number of proteins in hHEP keeps increasing while the other two are relatively stable?

This is indeed a surprising observation, which also intrigued us. This may be due to the different directions of proteome shift during primary cell culture of the different cell types (see also answer to the question 11). During culture of primary hepatocytes, the majority of the significantly changed proteins were down-regulated and they are related to metabolic processes (Fig 6d). Since metabolic enzymes comprise the top abundant proteins in hepatocytes, decrease in their abundance levels may reduce the dynamic range, therefore allowing more lowly abundant proteins to be detected. Conversely, the majority of the significantly changed proteins during culture of hepatic stellate cells are up-regulated, representing collagen fibril and extracellular matrix organization among others (Fig 6e). Since cytoskeletal framework components are among the top abundant proteins in hepatic stellate cells, a further increase in their

abundance can even increase the dynamic range, therefore reducing the chance of lowly abundant proteins to be detected.

14. Figure 6: What are the common proteins between the three cell types that changed over time?

Only eleven proteins that change over time were common between the three cell types, including enzymes (ALDOC, HMOX1, SMPD1, and ASS1), cellular structural proteins (KRT18, KRT19, and SMTN), immunity proteins (GLIPR2, HLA-B), autophagy receptor (SQSTM1), and kinase receptor (TGFBR1). While it is difficult to draw conclusions based on the very small number of commonly changed proteins between the three cell types, this may indicate that the proteome changes we observed for each cell type are not some general effects of cell culture but rather specific to each cell type.

15. Figure 6f: Do they authors observe any significant decrease of protein intensity?

Thank you for raising this question. As shown in Fig 6e, we also observed significant decrease of protein intensity (342 proteins, Dataset EV4). When doing functional annotation and enrichment analysis on these proteins (Enrichr, online), the following KEGG pathways were significantly enriched: “RNA transport”, “spliceosome”, “fatty acid biosynthesis”, “PPAR signaling pathway” and “ribosome biogenesis”. This could indicate a decrease in global protein synthesis rate and reduced proliferation upon culture and activation of HSC. It is not surprising to find decreased intensity in proteins related to fatty acid biosynthesis given that loss of lipid droplets is one of the two prominent features of HSC activation, which we also observed in microscopy when performing the in vitro cell culture experiment. PPARs are nuclear receptors that play a key role in the transcriptional regulation of glucose and lipid metabolism, and PPAR agonists have been investigated as possible therapeutic agents for liver disease. The expression of PPARγ is high in quiescent HSCs; however, PPARγ is suppressed during fibrosis process (Wu et al., 2020). Therefore, our observation of decreased protein levels in PPAR signaling confirms what is known but may also provide novel therapeutic targets, such as the proteins involved in lipid synthesis and degradation that we observed (FADS2, HMGCS1, FABP5, SCD, ACSL3). In the revised manuscript, we have incorporated the decreased proteins and their indication in liver disease on Page 12, Line 481-489 (line 472-480 clean version) to address this question.

“*In total, 342 proteins significantly decreased during primary culture of HSC (Dataset EV4). Among the significantly enriched KEGG pathways are ‘fatty acid biosynthesis’ and ‘PPAR signaling pathway’. Decreased intensity in proteins related to fatty acid biosynthesis may reflect the loss of lipid droplet, one of the two prominent features of HSC activation, which we also observed morphologically under the microscope. As mentioned above, PPAR agonists have been investigated as possible therapeutic agents for liver disease. The expression of PPARγ is high in quiescent HSCs; however, PPARγ is suppressed during fibrosis process (Wu et al., 2020). Therefore, our observation of decreased protein levels in PPAR signaling confirms what is known but may also provide novel therapeutic targets, such as the proteins involved in lipid synthesis and degradation (FADS2, HMGCS1, FABP5, SCD, ACSL3).*”

16. Figure 7: Reasonable number of samples in the cohort. Any confounder(s) regressed in the ANOVA (e.g., age, sex, 'obesity')?

Thank you for raising this very important point. In the revised version, we performed ANCOVA and corrected for age and sex. This resulted in 1,644 proteins being significantly different between the three groups (2,772 before). As a consequence, PDGFA in Figure 7d is not significant anymore, and the same is true for three of the 14 CYP450 enzymes in Figure 7e. In the revised manuscript, we have updated the results in Figure 7 and on Page 12, line 499-514 (line 490-505 clean version). We did not include BMI as a covariate because the three experimental groups have very different BMI (median 42, 50 and 25 for control,

NASH and cirrhosis group). Adding BMI as a covariate alone results in only 759 proteins being significant. While obesity and therefore higher BMI is associated with NASH development, in cirrhosis the patients have rather lower BMI, if the weight without ascites (fluid accumulation in the abdomen) is taken. Cachexia and sarcopenia (loss of weight and muscle mass) are clinical features of liver cirrhosis, especially when alcohol-related, due to various reasons including malnutrition. In this experimental design, adding BMI as a covariate will weaken our ability to detect proteome differences underlying the disease groups. Nonetheless, in the Jupyter notebook, we provided ANCOVA results correcting for age, sex and BMI, in order to render the database as transparent as possible.

17. Figure 7: The authors wrote: "These results provide the molecular basis for the clinical observations of liver cirrhosis, characterized by the replacement of normal liver tissue by scar tissue, the formation of new vessels leading to abnormal angioarchitecture in the cirrhotic liver, and the compromised immune system and dysregulated immune cell activation (Schierwagen et al, 2020; Trebicka et al, 2019)."

Did they observe an increase of the blood-related proteins? or proteins that might be considered as "specific to HepA or PorV"?

Thank you for this question. The summary was based on ontology enrichment analysis that we performed in which we found that the GOBP terms “angiogenesis” (39 proteins) and “sprouting angiogenesis” (six proteins) were significantly enriched in the upregulated proteins in cirrhotic liver biopsies, among other angiogenesis-related terms (Dataset EV5). Indeed, seven of the upregulated proteins in cirrhosis were uniquely detected in hepatic artery (HepA) or portal vein (PorV) as compared to bulk liver biopsy in our in-depth proteome atlas analysis, including two uniquely detected in both HepA and PorV: Tropomyosin alpha-1 chain (TPM1), Kinesin light chain 1 (KLC1). This may enhance the strength of the study as it allows, in some cases, the attribution of proteome changes observed in bulk liver biopsy to tissue types and even cell types. We now highlight these insights in the revised manuscript on Page 13, line 506-509 (line 497-500 clean version).

*“In particular, we found seven proteins that were significantly upregulated in cirrhotic liver biopsies to be uniquely detected in hepatic artery or portal vein in our in-depth proteome atlas analysis just above (Fig 2A). Of these, tropomyosin alpha-1 chain (TPM1) and Kinesin light chain 1 (KLC1) were detected in both but not in bulk liver.”*

**References for point-by-point answers**

Azimifar, S.B., Nagaraj, N., Cox, J., and Mann, M. (2014). Cell-type-resolved quantitative proteomics of murine liver. Cell metabolism *20*, 1076-1087.

Bekker-Jensen, D.B., Martínez-Val, A., Steigerwald, S., Rüther, P., Fort, K.L., Arrey, T.N., Harder, A., Makarov, A., and Olsen, J.V. (2020). A Compact Quadrupole-Orbitrap Mass Spectrometer with FAIMS Interface Improves Proteome Coverage in Short LC Gradients. Molecular & cellular proteomics : MCP *19*, 716-729.

Bilzer, M., Roggel, F., and Gerbes, A.L. (2006). Role of Kupffer cells in host defense and liver disease. Liver Int *26*, 1175-1186.

Hebert, A.S., Prasad, S., Belford, M.W., Bailey, D.J., McAlister, G.C., Abbatiello, S.E., Huguet, R., Wouters, E.R., Dunyach, J.-J., Brademan, D.R.*, et al.* (2018). Comprehensive Single-Shot Proteomics with FAIMS on a Hybrid Orbitrap Mass Spectrometer. Analytical chemistry *90*, 9529-9537.

Wang, Z.-Y., Keogh, A., Waldt, A., Cuttat, R., Neri, M., Zhu, S., Schuierer, S., Ruchti, A., Crochemore, C., Knehr, J.*, et al.* (2021). Single-cell and bulk transcriptomics of the liver reveals potential targets of NASH with fibrosis. Scientific reports *11*, 19396.

Wu, L., Guo, C., and Wu, J. (2020). Therapeutic potential of PPARγ natural agonists in liver diseases. Journal of cellular and molecular medicine *24*, 2736-2748.

## 1st Revision - Editorial Decision 27th Apr 2022

Thank you for sending us your revised manuscript. We have now evaluated your revised study. We think that the performed revisions have satisfactorily addressed the reviewers' concerns. I am glad to inform you that we can soon accept the study for publication, pending some minor editorial issues listed below.

## 2nd Authors' Response to Reviewers 27th Apr 2022

The authors have made all requested editorial changes.

## ACCEPTED 28th Apr 2022

Thank you again for sending us your revised manuscript. We are now satisfied with the modifications made and I am pleased to inform you that your paper has been accepted for publication.

## EMBO Press Author Checklist

| | |
|---|---|
| Corresponding Author Name: Jonel Trebicka and Matthias Mann | |
| Journal Submitted to: Molecular Systems Biology | |
| Manuscript Number: MSB-2022-10947 | |

**USEFUL LINKS FOR COMPLETING THIS FORM**
The EMBO Journal - Author Guidelines
EMBO Reports - Author Guidelines
Molecular Systems Biology - Author Guidelines
EMBO Molecular Medicine - Author Guidelines

**Reporting Checklist for Life Science Articles (updated January 2022)**
This checklist is adapted from Materials Design Analysis Reporting (MDAR) Checklist for Authors. MDAR establishes a minimum set of requirements in transparent reporting in the life sciences (see Statement of Task: 10.31222/osf.io/9sm4x). Please follow the journal's guidelines in preparing your manuscript.
**Please note that a copy of this checklist will be published alongside your article.**

**Abridged guidelines for figures**
**1. Data**
The data shown in figures should satisfy the following conditions:
- the data were obtained and processed according to the field's best practice and are presented to reflect the results of the experiments in an accurate and unbiased manner.
- ideally, figure panels should include only measurements that are directly comparable to each other and obtained with the same assay.
- plots include clearly labeled error bars for independent experiments and sample sizes. Unless justified, error bars should not be shown for technical replicates.
- if n<5, the individual data points from each experiment should be plotted. Any statistical test employed should be justified.
- Source Data should be included to report the data underlying figures according to the guidelines set out in the authorship guidelines on Data Presentation.

**2. Captions**
Each figure caption should contain the following information, for each panel where they are relevant:
- a specification of the experimental system investigated (eg cell line, species name).
- the assay(s) and method(s) used to carry out the reported observations and measurements.
- an explicit mention of the biological and chemical entity(ies) that are being measured.
- an explicit mention of the biological and chemical entity(ies) that are altered/varied/perturbed in a controlled manner.
- the exact sample size (n) for each experimental group/condition, given as a number, not a range;
- a description of the sample collection allowing the reader to understand whether the samples represent technical or biological replicates (including how many animals, litters, cultures, etc.).
- a statement of how many times the experiment shown was independently replicated in the laboratory.
- definitions of statistical methods and measures:
  - common tests, such as t-test (please specify whether paired vs. unpaired), simple χ2 tests, Wilcoxon and Mann-Whitney tests, can be unambiguously identified by name only, but more complex techniques should be described in the methods section;
  - are tests one-sided or two-sided?
  - are there adjustments for multiple comparisons?
  - exact statistical test results, e.g., P values = x but not P values < x;
  - definition of 'center values' as median or average;
  - definition of error bars as s.d. or s.e.m.

**Please complete ALL of the questions below.**
**Select "Not Applicable" only when the requested information is not relevant for your study.**

**Materials**

| Newly Created Materials | Information included in the manuscript? | In which section is the information available? (Reagents and Tools Table, Materials and Methods, Figures, Data Availability Section) |
|---|---|---|
| New materials and reagents need to be available; do any restrictions apply? | Select response | Data Availability Section |

| Antibodies | Information included in the manuscript? | In which section is the information available? (Reagents and Tools Table, Materials and Methods, Figures, Data Availability Section) |
|---|---|---|
| For **antibodies** provide the following information:<br>- Commercial antibodies: RRID (if possible) or supplier name, catalogue number and or/clone number<br>- Non-commercial: RRID or citation | Select response | Not Applicable |

| DNA and RNA sequences | Information included in the manuscript? | In which section is the information available? (Reagents and Tools Table, Materials and Methods, Figures, Data Availability Section) |
|---|---|---|
| **Short novel DNA or RNA including primers, probes**: provide the sequences. | Select response | Not Applicable |

| Cell materials | Information included in the manuscript? | In which section is the information available? (Reagents and Tools Table, Materials and Methods, Figures, Data Availability Section) |
|---|---|---|
| **Cell lines:** Provide species information, strain. Provide accession number in repository **OR** supplier name, catalog number, clone number, and/**OR** RRID. | Select response | Reagents and Tools Table |
| **Primary cultures:** Provide species, strain, sex of origin, genetic modification status. | Select response | Reagents and Tools Table, Materials and Methods |
| Report if the cell lines were recently **authenticated** (e.g., by STR profiling) and tested for mycoplasma contamination. | Select response | Not Applicable |

| Experimental animals | Information included in the manuscript? | In which section is the information available? (Reagents and Tools Table, Materials and Methods, Figures, Data Availability Section) |
|---|---|---|
| **Laboratory animals or Model organisms:** Provide species, strain, sex, age, genetic modification status. Provide accession number in repository **OR** supplier name, catalog number, clone number, **OR** RRID. | Select response | Not Applicable |
| **Animal observed in or captured from the field:** Provide species, sex, and age where possible. | Select response | Not Applicable |
| Please detail **housing and husbandry conditions**. | Select response | Not Applicable |

| Plants and microbes | Information included in the manuscript? | In which section is the information available? (Reagents and Tools Table, Materials and Methods, Figures, Data Availability Section) |
|---|---|---|
| **Plants:** provide species and strain, ecotype and cultivar where relevant, unique accession number if available, and source (including location for collected wild specimens). | Select response | Not Applicable |
| **Microbes:** provide species and strain, unique accession number if available, and source. | Select response | Not Applicable |

| Human research participants | Information included in the manuscript? | In which section is the information available? (Reagents and Tools Table, Materials and Methods, Figures, Data Availability Section) |
|---|---|---|
| If collected and within the bounds of privacy constraints report on age, sex and gender or ethnicity for all study participants. | Select response | Materials and Methods |

| Core facilities | Information included in the manuscript? | In which section is the information available? (Reagents and Tools Table, Materials and Methods, Figures, Data Availability Section) |
|---|---|---|
| If your work benefited from core facilities, was their service mentioned in the acknowledgments section? | Select response | Not Applicable |

**Design**

| Study protocol | Information included in the manuscript? | In which section is the information available? (Reagents and Tools Table, Materials and Methods, Figures, Data Availability Section) |
|---|---|---|
| If study protocol has been **pre-registered, provide DOI in the manuscript**. For clinical trials, provide the trial registration number **OR** cite DOI. | Select response | Not Applicable |
| Report the **clinical trial registration number** (at ClinicalTrials.gov or equivalent), where applicable. | Select response | Not Applicable |

| Laboratory protocol | Information included in the manuscript? | In which section is the information available? (Reagents and Tools Table, Materials and Methods, Figures, Data Availability Section) |
|---|---|---|
| Provide DOI OR other citation details if **external detailed step-by-step protocols** are available. | Select response | Materials and Methods |

| Experimental study design and statistics | Information included in the manuscript? | In which section is the information available? (Reagents and Tools Table, Materials and Methods, Figures, Data Availability Section) |
|---|---|---|

| | | |
|---|---|---|
| Include a statement about **sample size** estimate even if no statistical methods were used. | Select response | Not Applicable |
| Were any steps taken to minimize the effects of subjective bias when allocating animals/samples to treatment (e.g. **randomization procedure**)? If yes, have they been described? | Select response | Materials and Methods |
| Include a statement about **blinding** even if no blinding was done. | Select response | Materials and Methods |
| Describe **inclusion/exclusion criteria** if samples or animals were excluded from the analysis. Were the criteria pre-established?<br><br>If sample or data points were omitted from analysis, report if this was due to attrition or intentional exclusion and provide justification. | Select response | Materials and Methods |
| For every figure, are **statistical tests** justified as appropriate? Do the data meet the assumptions of the tests (e.g., normal distribution)? Describe any methods used to assess it. Is there an estimate of variation within each group of data? Is the variance similar between the groups that are being statistically compared? | Select response | Materials and Methods |

| Sample definition and in-laboratory replication | Information included in the manuscript? | In which section is the information available?<br>(Reagents and Tools Table, Materials and Methods, Figures, Data Availability Section) |
|---|---|---|
| In the figure legends: state number of times the experiment was **replicated** in laboratory. | Select response | Figures |
| In the figure legends: define whether data describe **technical or biological replicates**. | Select response | Figures |

## Ethics

| Ethics | Information included in the manuscript? | In which section is the information available?<br>(Reagents and Tools Table, Materials and Methods, Figures, Data Availability Section) |
|---|---|---|
| Studies involving **human participants**: State details of **authority granting ethics approval** (IRB or equivalent committee(s), provide reference number for approval. | Select response | Materials and Methods |
| Studies involving **human participants**: Include a statement confirming that **informed consent** was obtained from all subjects and that the experiments conformed to the principles set out in the WMA Declaration of Helsinki and the Department of Health and Human Services Belmont Report. | Select response | Materials and Methods |
| Studies involving **human participants:** For publication of **patient photos**, include a statement confirming that consent to publish was obtained. | Select response | Not Applicable |
| Studies involving experimental **animals**: State details of **authority granting ethics approval** (IRB or equivalent committee(s), provide reference number for approval. Include a statement of compliance with ethical regulations. | Select response | Not Applicable |
| Studies involving **specimen and field samples:** State if relevant **permits** obtained, provide details of authority approving study; if none were required, explain why. | Select response | Materials and Methods |

| Dual Use Research of Concern (DURC) | Information included in the manuscript? | In which section is the information available?<br>(Reagents and Tools Table, Materials and Methods, Figures, Data Availability Section) |
|---|---|---|
| Could your study fall under dual use research restrictions? Please check biosecurity documents and list of **select agents and toxins** (CDC): https://www.selectagents.gov/sat/list.htm | Select response | Not Applicable |
| If you used a select agent, is the security level of the lab appropriate and reported in the manuscript? | Select response | Not Applicable |
| If a study is subject to dual use research of concern regulations, is the name of the **authority granting approval and reference number** for the regulatory approval provided in the manuscript? | Select response | Not Applicable |

## Reporting

The MDAR framework recommends adoption of discipline-specific guidelines, established and endorsed through community initiatives. Journals have their own policy about requiring specific guidelines and recommendations to complement MDAR.

| Adherence to community standards | Information included in the manuscript? | In which section is the information available?<br>(Reagents and Tools Table, Materials and Methods, Figures, Data Availability Section) |
|---|---|---|
| State if relevant guidelines or checklists (e.g., **ICMJE, MIBBI, ARRIVE, PRISMA**) have been followed or provided. | Select response | Not Applicable |
| For **tumor marker prognostic studies,** we recommend that you follow the **REMARK** reporting guidelines (see link list at top right). See author guidelines, under 'Reporting Guidelines'. Please confirm you have followed these guidelines. | Select response | Not Applicable |
| For **phase II and III randomized controlled trials**, please refer to the **CONSORT** flow diagram (see link list at top right) and submit the CONSORT checklist (see link list at top right) with your submission. See author guidelines, under 'Reporting Guidelines'. Please confirm you have submitted this list. | Select response | Not Applicable |

## Data Availability

| Data availability | Information included in the manuscript? | In which section is the information available?<br>(Reagents and Tools Table, Materials and Methods, Figures, Data Availability Section) |
|---|---|---|
| Have **primary datasets** been deposited according to the journal's guidelines (see 'Data Deposition' section) and the respective accession numbers provided in the Data Availability Section? | Select response | Data Availability Section |
| Were **human clinical and genomic datasets** deposited in a public access-controlled repository in accordance to ethical obligations to the patients and to the applicable consent agreement? | Select response | Not Applicable |
| Are **computational models** that are central and integral to a study available without restrictions in a machine-readable form? Were the relevant accession numbers or links provided? | Select response | Data Availability Section |
| If publicly available data were reused, provide the respective **data citations in the reference list**. | Select response | Materials and Methods |
